# Nitrogen-Containing Diterpenoids, Sesquiterpenoids, and Nor-Diterpenoids from *Cespitularia* *taeniata*

**DOI:** 10.3390/md13095796

**Published:** 2015-09-15

**Authors:** Shih-Sheng Wang, Yuan-Bin Cheng, Yu-Chi Lin, Chia-Ching Liaw, Jiun-Yang Chang, Yao-Haur Kuo, Ya-Ching Shen

**Affiliations:** 1 School of Pharmacy, College of Medicine, National Taiwan University, Taipei 100, Taiwan; E-Mails: aska@newbellus.com.tw (S.-S.W.); z10108042@email.ncku.edu.tw (Y.-C.L.); biogodas@hotmail.com (C.-C.L.); 2 Department of Marine Biotechnology and Resources, National Sun Yat-Sen University, Kaohsiung 804, Taiwan; E-Mail: dryang323@gmail.com; 3 Graduate Institute of Natural Products, College of Pharmacy, Kaohsiung Medical University, Kaohsiung 807, Taiwan; E-Mail: jmb@kmu.edu.tw; 4 National Research Institute of Chinese Medicine, Taipei 112, Taiwan; E-Mail: kuoyh@nricm.edu.tw

**Keywords:** *Cespitularia taeniata*, verticillene diterpenoids, cytotoxicity

## Abstract

Two new nitrogen-containing verticillene diterpenoids, cespilamides A and B (**1** and **2**), three new nitrogen-containing sesquiterpenoids, cespilamides C–E (**3**–**5**), and five new norverticillene and verticillene diterpenoids, cespitaenins A–E (**6**–**10**), were isolated from the Taiwanese soft coral *Cespitularia taeniata*. Compound **1** possesses an unusual oxazo ring system at C-10 while compound **2** displays an unprecedented C–C bond cleavage between C-10 and C-11 with an *N*-ethylphenyl group at C-10. Biogenetic pathways of **1** and **2** are proposed. The absolute configuration of **1** was confirmed by Mosher’s method and molecular mechanics calculations (MM2). The cytotoxicities of compounds **1**–**10** were evaluated against a small panel of human cancer cell lines.

## 1. Introduction

**Figure 1:**
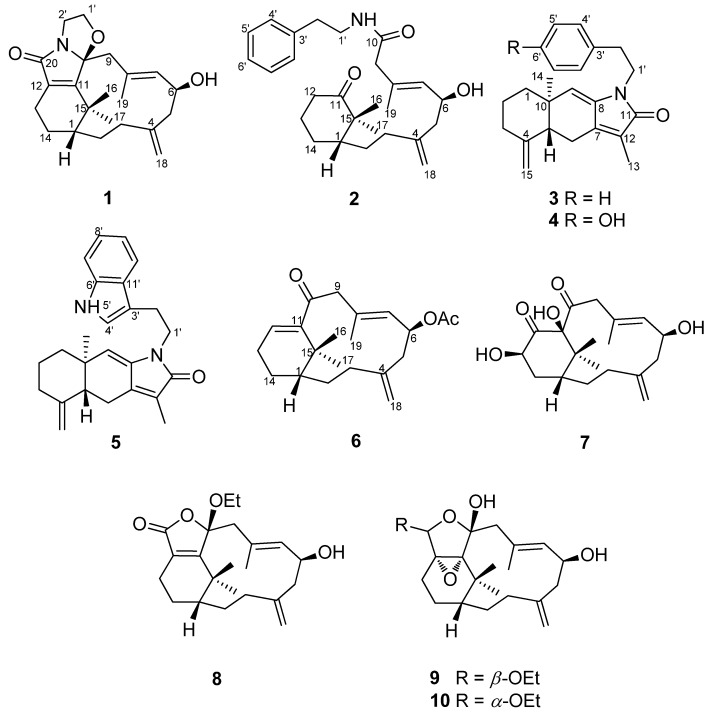
Structures of metabolites **1**–**10**.

Marine invertebrates have been proven to secrete a number of secondary metabolites for self-defense, and those marine natural products usually show unexpected bioactivities. For example, sarcodictyins isolated from *Bellonella albiflora* and eleutherobins obtained from *Eleutherobia aurea* showed significant cytotoxicities [1,2]. Aberrarone discovered from gorgonian *Pseudopterogorgia elisabethae* possessed potent antibacterial effects [3]. Those compounds can benefit new drug development and also inspire drug design. Soft corals of the genus *Cespitularia* produce various types of terpenoids such as cembranes, neodolabellanes, cespitularanes, and verticillanes [4,5,6,7,8]. These compounds are reported to demonstrate cytotoxic and immune-modulatory activities [9,10,11,12,13,14]. In our continuous research of Taiwanese soft corals, a series of nor-verticillenes and nitrogen-containing verticillanes from *C. taeniata* were isolated and reported [10,11]. Those findings impel us to further investigate this benthos. In this paper, we describe the isolation and structural elucidation of ten new marine natural products including two nitrogen-containing verticillanes (**1** and **2**), three nitrogen-containing sesquiterpenes (**3**–**5**), two norverticillanes (**6** and **7**), and three verticillanes (**8**–**10**) from Taiwanese soft coral *C. taeniata* (Figure 1).

## 2. Results and Discussion

The EtOAc-MeOH (1:1) extract of *C*. *taeniata* was partitioned between H_2_O and EtOAc to give an EtOAc-soluble fraction. Extensive column chromatography and HPLC purification allowed the separation of ten new compounds (**1**–**10**).

**Figure 2:**
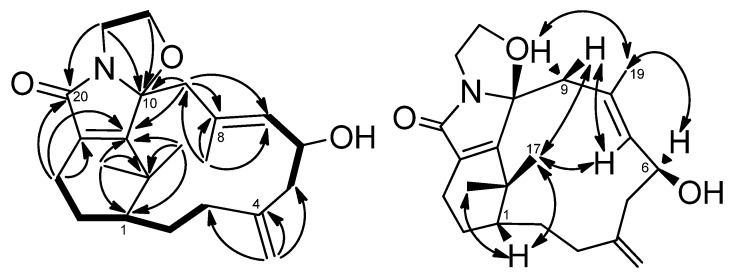
COSY (bold bond), HMBC (arrow) and selected NOESY correlations of **1**.

**Figure 3:**
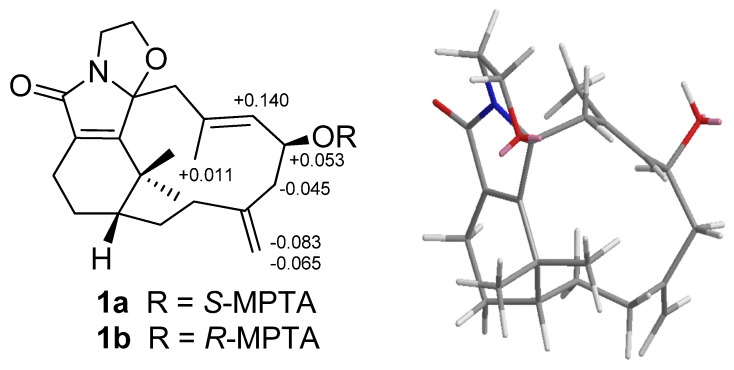
Mosher reaction products (**1a**, **1b**), Data are difference values of Δ*_S_*_-*R*_ (ppm); and computer-generated perspective model of **1**.

Cespilamide A (**1**), ${\left[\alpha \right]}_{\text{D}}^{25}$ −118.2 (CH_2_Cl_2_), had a molecular formula of C_22_H_31_O_3_N as deduced from the NMR and HRESIMS (*m*/*z* 358.2380 [M + Na]^+^, calcd. 358.2382) data, indicating eight indices of hydrogen deficiency. The IR spectrum revealed the presence of hydroxy (3421 cm^−1^) and conjugated amide (1695 cm^−1^) moieties. The ^1^H and ^13^C-NMR data (Table 1 and Table 2) showed the presence of an amidocarbonyl (δ_C_ 177.1), a trisubstituted olefinic unit [δ_C_ 134.8 (s), 132.3 (d); δ_H_ 5.50, d, *J* = 8.0 Hz], a tetrasubstituted olefinic moity (δ_C_ 166.2, 133.5), and an exomethylene group [δ_C_ 146.2 (s), 114.2 (t); δ_H_ 4.87, 4.83, each brs]. The DEPT NMR spectrum indicated an oxygenated quarternary carbon (δ_C_ 102.8), an oxygenated methine carbon (δ_C_ 68.5 d), eight methylene carbons (δ_C_ 17.9, 24.4, 32.3, 33.0, 42.5, 43.8, 46.6, 68.8), and three methyl groups (δ_H_ 1.47, 1.19, 1.59, each 3H and s; δ_C_ 17.2, 34.3, 35.2). The ^1^H–^1^H COSY experiment (Figure 2) showed three sets of correlations, H-1′′/H-2′′, H-7/H-6/H-5 and H-3/H-2/H-1/H-14/H-13, and the latter two sets of proton sequences were further connected by the HMBC correlations (Figure 2) of H-18/C-3 (δ_C_ 33.0), C-4 (δ_C_ 146.2), and C-5 (δ_C_ 43.8). Furthermore, the HMBC correlations of CH_3_-16, CH_3_-17/C-15 (δ_C_ 37.9), C-1 (δ_C_ 43.5), C-11 (δ_C_ 166.2) and H-13/C-12 (δ_C_ 133.5), C-20 (δ_C_ 177.1), C-11 indicated that compound **1** possesses a 2′,2′-dimethylcyclohexene moiety. The HMBC correlations of H-9/C-7 (δ_C_ 134.8), C-8 (δ_C_ 132.3), C-10 (δ_C_ 102.8), C-11 and CH_3_-19/C-7, C-8, C-9 (δ_C_ 46.6) were used to establish the planar structure of compound **1**, except for the C1′-C2′ moiety. Comparison of the ^1^H- and ^13^C-NMR data of **1** with those of cespitulactam D revealed that they have similar verticillene skeletons [12]. ^1^H–^1^H COSY correlations of H-1′ (δ_H_ 3.84, m; 4.11, m)/H-2′ (δ_H_ 3.27, m; 3.90, m) and HMBC correlations of H-2′/C-10, C-20 and H-1′/C-10, C-11 suggested that there is an ethylene moiety between the C-10 oxygen function and the nitrogen of the amide moiety. The configuration of compound **1** was determined by NOESY correlations and the Mosher’s ester method. It was assumed that compound **1** has the same absolute configuration at C-1 as naturally-occurring verticillene diterpenoids, such as cespitulactams, cespitularines, and toxoids [10,12,13]. NOESY (Figure 2) correlations of H-1/Me-16, Me-17 and H-7/ Me-17 indicated the β-orientation of Me-16 and Me-17. Moreover, NOESY correlations of H-6/Me-19/H-9α (δ_H_ 2.83) and H-7/H-9β (δ_H_ 2.58) suggested that H-6 is α-oriented. The configuration of the hydroxy group at C-6 was further determined by Mosher’s reactions to yield products **1a** and **1b**. The results, illustrated in Figure 3, suggested that C-6 has the *S* configuration. A computer-generated MM2 structure for compound **1** calculated for the lowest energy is illustrated in Figure 3. The result also agreed with a *S* configuration at C-6. Due to lack of NOE interaction between H-7 and Me-19, the geometry of the 7,8-double bond in **1** was deduced to be *E.*

**Table 1:** ^1^H-NMR data for compounds **1**–**10** *^a^*.

Position	1 *^b^*	2 *^c^*	3 *^b^*	4 *^b^*	5 *^b^*	6 *^b^*	7 *^b^*	8 *^b^*	9 *^c^*	10 *^b^*
1	1.59, m	1.44, m	1.39, m	1.59, m	1.32, m	1.66, m	2.18, m	1.60, m	1.46, m	1.43, m
			1.46, m		1.43, m					
2	1.54, m	1.25, m	1.56, m	1.66, m	1.61, m	1.50, m	1.12, m	2.24, m	2.30, m	2.27, m
		1.62, m				1.98, m				
3	2.11, m	1.97, m	2.01, m	2.36, m	2.00, m	2.68, m	1.93, m	2.15, m	2.08, m	2.13, m
	2.30, m	2.13, m	2.33, m		2.31, m		2.25, m		2.18, m	
5	2.38, m	2.19, m	2.18, m	2.18, m	2.11, m	2.28, m	2.73, dd (3.9, 12.6)	2.40, m	2.23, m	2.20, m
						2.50, m			2.65, m	2.60, m
6	4.37, m	4.44, dt (5.5, 8.5)	2.42, m	2.63, m	2.36, m	5.38, dt (8.4, 2.4)	4.55, dt (3.9, 9.6)	4.36, dt (3.9, 7.8)	4.50, dt (3.0, 8.5)	4.40, dt (3.0, 8.7)
			2.60, m		2.57, m					
7	5.50, d (8.0)	5.28, d (8.5)				5.15, d (8.4)	5.56, d (9.3)	5.51, d (7.8)	5.45, d (8.5)	5.43, d (8.7)
9	2.58, d (13.8)	2.89, s	5.05, s	5.14, s	5.06, s	3.07, d (15.9)	2.84, d (13.5)	2.85, d (14.1)	2.51, d (14.5)	2.53, d (14.4)
	2.83, d (13.8)					3.40, d (15.9)	3.89, d (13.5)	3.02, d (14.1)	3.02, d (14.5)	3.01, d (14.4)
12		2.31, m				6.20, t (3.6)				
		2.50, m								
13	1.63, m	1.99, m	1.87, s	1.87, s	1.88, s	2.31, m	4.39, t (3.3)	1.47, m	1.59, m	1.63, m
	2.15, m								1.69, m	
14	2.15, m	1.88, m	0.80, s	0.84, s	0.73, s	2.25, m	2.14, m	1.66, m	1.08, m	1.16, m
	2.35, m							2.20, m	1.86, m	1.92, m
15			4.61, s	4.61, s	4.59, s					
			4.86, s	4.87, s	4.85, s					
16	1.47, s	1.11, s				1.27, s	0.77, s	1.24, s	0.94, s	0.97, s
17	1.19, s	1.03, s				1.20, s	1.47, s	1.44, s	1.32, s	1.31, s
18	4.83, br s	4.81, s				4.80, s	4.92, s	4.83, s	4.92, s	4.92, s
	4.82, br s	4.86, s				4.77, s	4.96, s	4.84, s	4.92, s	4.92, s
19	1.59, s	1.67, s				1.76, s	1.89, s	1.56, s	1.82, s	1.84, s
20									4.46, s	4.56, s
1′	3.84, m	3.51, m	3.74, t (7.5)	3.72, t (7.5)	3.84, dt (7.2, 14.4)			3.43, m	3.50, m	3.56, m
	4.11, m							3.63, m	3.86, m	3.77, m
2′	3.27, m	2.81, t (6.5)	2.86, t (7.5)	2.78, t (7.5)	3.03, t (7.2)			1.20, t (6.9)	1.24, t (7.0)	1.15, t (6.9)
	3.90, m									
4′		7.18, d (7.0)	7.17, d (6.6)	7.01, d (8.4)	7.02, d (1.5)					
5′		7.22, t (7.0)	7.19, t (6.6)	6.74, d (8.4)	8.05, (N H)					
6′		7.31, t (7.0)	7.26, t (6.6)							
7′		7.22, t (7.0)	7.19, t (6.6)	6.74, d (8.4)	7.35, d (7.8)					
8′		7.18, d (7.0)	7.17, d (6.6)	7.01, d (8.4)	7.18, t (7.2)					
9′					7.10, t (7.2)					
10′					7.59, d (7.8)					
OAc						2.01 s				

*^a^* Chemical shifts are in ppm; *J* values (Hz) are in parentheses. *^b^* Recorded in CDCl_3_ at 300 MHz. *^c^* Recorded in CDCl_3_ at 500 MHz.

**Table 2:** ^13^C-NMR data for compounds **1**–**10** *^a^.*

Position	1 *^b^*	2 *^c^*	3 *^b^*	4 *^b^*	5 *^b^*	6 *^b^*	7 *^b^*	8 *^b^*	9 *^c^*	10 *^b^*
1	43.5 d	47.1 d	39.5 t	39.5 t	39.2 t	43.1 d	46.8 d	44.0 d	44.2 d	44.5 d
2	32.3 t	27.7 t	23.2 t	23.2 t	23.1 t	30.6 t	32.9 t	17.6 t	26.2 t	25.4 t
3	33.0 t	34.3 t	36.3 t	36.3 t	36.2 t	31.3 t	39.2 t	33.6 t	37.8 t	37.9 t
4	146.2 s	145.7 s	148.6 s	148.8 s	148.8 s	146.4 s	144.8 s	145.9 s	145.8 s	147.2 s
5	43.8 t	43.9 t	48.9 d	49.0 d	48.8 d	41.1 t	46.8 t	43.7 t	45.8 t	47.1 t
6	68.5 d	65.8 d	22.3 t	22.3 t	22.1 t	72.3 d	70.2 d	68.2 d	69.2 d	69.2 d
7	134.8 d	132.5 d	139.8 s	139.8 s	140.0 s	129.0 d	132.7 d	135.6 d	133.2 d	134.1 d
8	132.3 s	132.9 s	137.1 s	137.3 s	137.2 s	133.3 s	133.2 s	131.4 s	132.8 s	131.1 s
9	46.6 t	47.5 d	119.1 d	119.6 d	119.1 d	50.7 t	49.4 t	47.0 t	41.0 t	41.3 t
10	102.8 s	170.2 s	38.7 s	37.9 s	37.5 s	202.1 s	208.1 s	110.9 s	94.2 s	93.0 s
11	166.2 s	216.0 s	170.0 s	171.1 s	170.2 s	148.0 s	92.2 s	166.6 s	72.8 s	72.4 s
12	133.5 s	37.8 t	123.9 s	124.2 s	124.1 s	135.4 d	214.5 s	129.5 s	78.0 s	79.1 s
13	24.4 t	25.0 t	8.4 q	8.4 q	8.4 q	23.8 t	74.8 d	32.1 t	31.6 t	26.0 t
14	17.9 t	25.9 t	18.6 q	18.6 q	18.3 q	22.8 t	24.3 t	24.4 t	33.9 t	34.4 t
15	37.9 s	48.9 s	107.0 t	107.1 t	106.8 t	35.4 s	46.8 s	37.4 s	37.6 s	37.5 s
16	35.2 q	22.8 q				32.8 q	25.8 q	33.7 q	25.1 q	25.0 q
17	34.3 q	19.9 q				24.8 q	26.5 q	24.5 q	26.0 q	26.1 q
18	114.2 t	113.0 t				113.5 t	115.5 t	114.5 t	115.6 t	114.0 t
19	17.2 q	16.7 q				19.5 q	17.6 q	17.1 q	17.3 q	16.5 q
20	177.1 s							170.5 s	103.5 d	107.3 d
1′	68.8 t	40.5 t	41.0 t	41.1 t	39.9 t			58.8 t	65.2 t	65.4 t
2′	42.5 t	35.2 t	35.4 t	34.3 t	24.8 t			15.1 q	15.0 q	14.8 q
3′		138.7 s	139.2 s	130.8 s	113.3 s					
4′		128.7 d	128.9 d	130.0 d	121.9 d					
5′		126.5 d	126.4 d	115.4 d						
6′		128.6 d	128.5 d	154.6 s	124.7 s					
7′					111.1 d					
8′					121.9 d					
9′					119.3 d					
10′					118.6 d					
11′					127.6 s					
OAc						170.1 s				
						21.3 q				

*^a^* Multiplicities (s = C, d = CH, t = CH_2_, q = CH_3_) and assignments made by HMQC and HMBC techniques. *^b^* Recorded in CDCl_3_ at 75 MHz. *^c^* Recorded in CDCl_3_ at 125 MHz.

**Figure 4:**
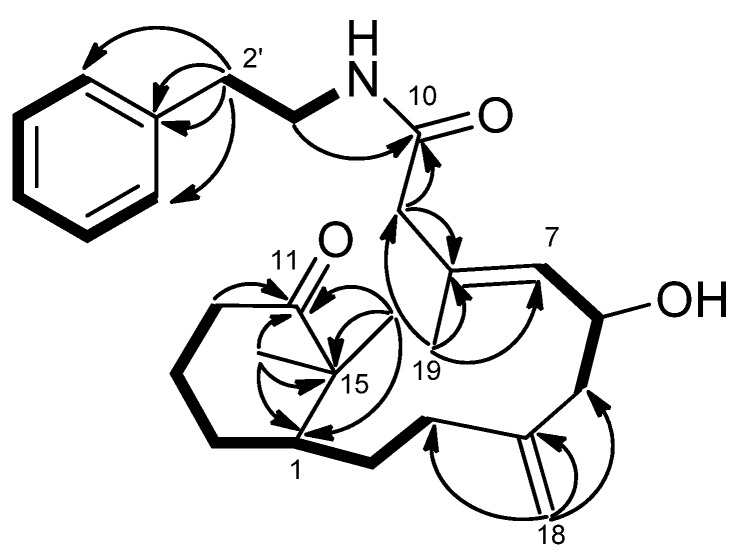
COSY (bold bond) and HMBC (arrow) correlations of **2**.

**Figure 5:**
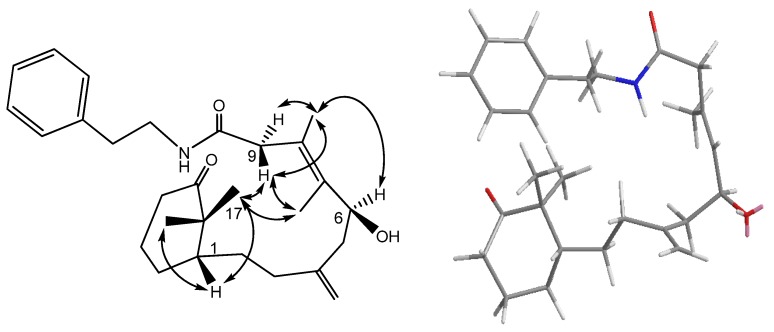
Selected NOESY correlations and computer-generated perspective model of **2**.

Cespilamide B (**2**), ${\left[\alpha \right]}_{\text{D}}^{25}$ −8.0 (CH_2_Cl_2_), was assigned a molecular formula of C_27_H_39_O_3_N, as deduced from the HRESIMS (*m*/*z* 448.2825 [M + Na]^+^, calcd. 448.2827), indicating nine indices of hydrogen deficiency. The presence of hydroxy, amide, and benzyl functionalities was indicated by IR absorptions at 3371, 1701, and 1647 cm^−1^. The ^1^H and ^13^C-NMR spectra revealed the presence of a ketocarbonyl (δ_C_ 216.0), an amide carbonyl (δ_C_ 170.2), a trisubstituted olefin [δ_C_ 132.9 (s), 132.5 (d); δ_H_ 5.28, d, *J* = 8.5 Hz], a 1,1-disubstituted olefin (δ_C_ 145.7) with an exomethylene group (δ_C_ 113.0; δ_H_ 4.86, 4.81, each s), an oxygenated methine carbon (δ_C_ 65.8), and a phenyl group [δ_C_ 138.7 (s), 128.7 (d, 2C), 126.5 (d, 2C), 128.6 (d); δ_H_ 7.18, d, *J* = 7.0 Hz (2H), δ_H_ 7.22 t, *J* = 7.0 Hz, δ_H_ 7.31 t, *J* = 7.0 Hz (2H)]. Thus, eight degrees of unsaturation were counted, leaving one further ring to be elucidated. The ^1^H–^1^H COSY (Figure 4) correlations of H-7/H-6/H-5, H-3/H-2/H-1/H-14/H-13/H-12, NH (δ_H_ 5.71, brs)/H-1′/H-2′ and H-4′/H-5′/H-6′/H-7′/H-8′ revealed the sequences of three fragments including H-5 to H-7, H-3 to H-12 and a benzylethyl amine side chain. The HMBC correlations (Figure 4) of H-9/C-10, C-8, H-12/C-11, Me-16/C-11, Me-17/C-11 and H-1′/C-10 permitted assignment of the two carbonyls at C-10 and C-11. Also, it established the connectivity between C-10 and C-1′. The absence of HMBC correlations between H-9/C-11, and H-12/C-10 indicated that compound **2** represents an unusual C-20 norditerpenoid [13] with bond cleavage between C-10 and C-11. The relative configuration of compound **2** was determined by NOESY experiments (Figure 5) and computer-generated perspective models using the MM2 force field calculation. A NOESY correlation between Me-19 and H-6, and the lack of a correlation between Me-19 and H-7 suggested that the 7,8-double bond has an *E* geometry, similar to compound **1**.

**Figure 6:**
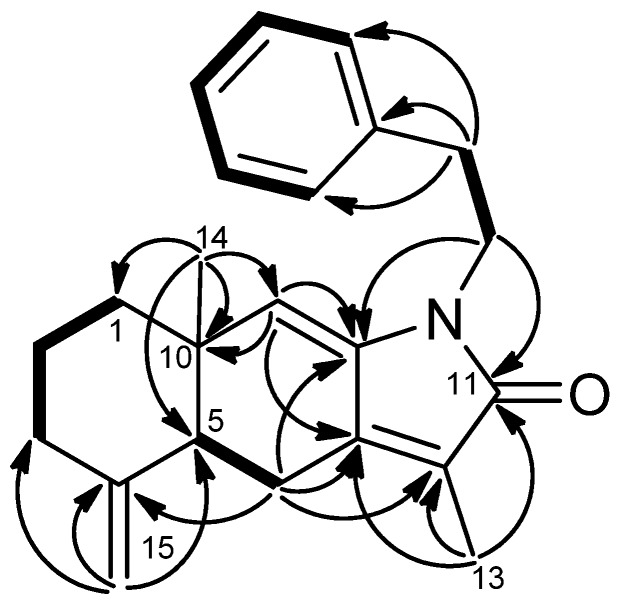
COSY (bold bond), HMBC (arrow) correlations of **3**.

**Figure 7:**
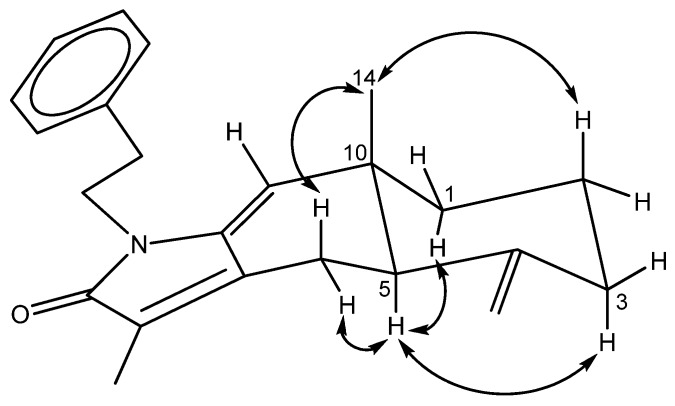
Selected NOESY correlation of **3**.

The HRESIMS determined the molecular formula of compound **3** as C_23_H_27_ON (*m*/*z* 356.1992 [M + Na]^+^, calcd. 356.1990) and indicated eleven degrees of unsaturation. The IR absorption of 1676 cm^−1^ suggested the presence of a conjugated amide group. The ^1^H, ^13^C (Table 1 and Table 2) and DEPT NMR spectroscopic data revealed the presence of an amide carbonyl (δ_C_ 170.0), a trisubstituted olefin [δ_C_ 137.1 (s), 119.1 (d); δ_H_ 5.05, s], an exomethylene group [δ_C_ 148 (s), 107.0 (t); δ_H_ 4.86, 4.61, each s], a tetrasubstituted olefin (δ_C_ 123.9, 139.8), a phenyl group [δ_C_ 139.2 (s), 128.9 (d, 2C), 126.4 (d, 2C), 128.5 (d); δ_H_ 7.17, d, *J* = 6.6 Hz (2H), δ_H_ 7.19, t, *J* = 6.6 Hz (2H), δ_H_ 7.26, t, *J* = 6.6 Hz], an aliphatic CH group (δ_H_ 2.18, m; δ_C_ 48.9), and four aliphatic CH_2_ group (δ_C_ 39.5, 23.2, 36.2, 22.3). The above findings accounted for five of the eight degrees of unsaturation, indicating that compound **3** is a tricyclic sesquiterpene with a phenyl group. ^1^H–^1^H COSY spectrum of **3** showed four sets of correlations, H-1/H-2/H-3, H-5/H-6, H-1′/H-2′, and H-4′/ H-5′/ H-6′/ H-7′/ H-8′. The HMBC correlations (Figure 6) of H_2_-15/C-2, C-4, C-5 confirmed an exocyclic double bond between C-3 and C-5. The HMBC correlations of CH_3_-13/C-12, C-11, C-7; Me-14/C-10, C-1, C-9, C-5, and H-9/C-10, C-8, C-7 not only suggested the occurrence of double bonds between C-7/C-12 and C-8/C-9 but also assign the methyl group at C-10 and C-12. The presence of an α,β-unsaturated δ-lactam was inferred from the IR and HMBC spectra. Moreover, the HMBC correlations of H-1′/C-11, C-8 and H-2′/C-3′, C-4′, C-8′ indicated an amide carbonyl at C-11 and a phenylethyl side chain attached to a nitrogen atom. The relative configuration of **3** was determined on the basis of NOESY experiment and comparison with the optical rotation and NMR data of recent published compounds, taenialactams A and B, which were isolated from *C. taeniata* [14]. Assuming that H-5 possesses an α-orientation similar to that of taenialactams, the lack of NOESY correlation between H-5 and Me-14, suggested that Me-14 is β-oriented (Figure 7).

The molecular formula of **4** was determined to be C_23_H_27_O_2_N (Δ = 11) by HRESIMS data (*m*/*z* 372.1937 [M + Na]^+^, calcd. 372.1939). The IR spectrum revealed the presence of hydroxy (3421 cm^−1^) and α,β-unsaturated γ-lactam (1695 cm^−1^) moieties. The ^1^H and ^13^C NMR spectra (Table 1 and Table 2) of compound **4** were similar to those of **3**, suggesting structural similarity with the exception that compound **4** contains a *para*-hydroxyphenylethyl side chain [δ_H_ 7.01, d, *J* = 8.4 Hz (2H), 6.74, d, *J* = 8.4 Hz (2H); δ_C_ 154.6 (s), 130.8 (s), 130.0 (d), 115.4 (d), 41.0 (t), 34.3 (t)] on the nitrogen atom, rather than a phenylethyl group as found in compound **3**. Interpretation of ^1^H–^1^H COSY and HMBC spectra of compound **4** also indicated the presence of a hydroxy group at C-6′. The relative configuration of compound **4** was determined by comparison with the NMR and the optical rotation of compound **3**.

**Figure 8:**
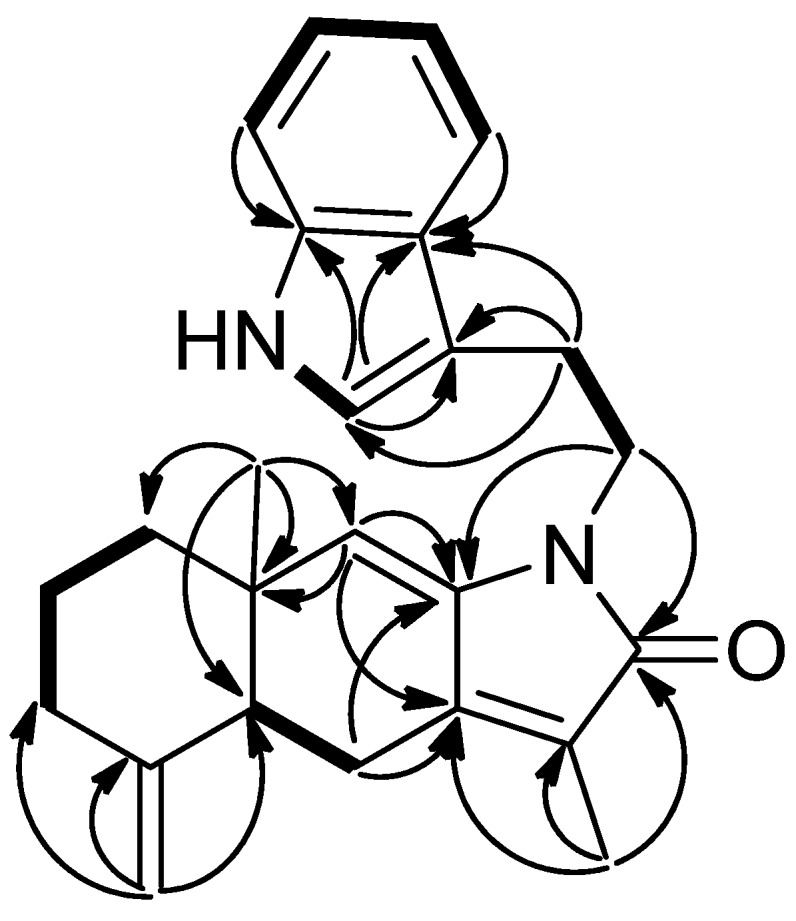
COSY (bold bond) and HMBC (arrow) correlations of **5**.

The molecular formula of compound **5** was shown to be C_25_H_28_ON_2_ (Δ = 13), as deduced from HRESIMS at *m*/*z* 395.2099 ([M + Na]^+^, calcd. 395.2099). Spectroscopic data of compound **5** were found to be similar to those of **3** and **4** except for the evidence of an ethylindole moiety. The LRMS of compound **5** exhibited a peak at *m*/*z* 229 [M + H − C_10_H_10_N]^+^, also consistent with the presence of an ethylindole group. In the ^1^H and ^13^C NMR spectra (Table 1 and Table 2), signals for a 3-ethylindole group [δ_H_ 3.84, dt, *J* = 14.4, 7.2 Hz, 3.03, t, *J* = 7.2 Hz, 7.02, d, *J* = 1.5 Hz, 7.35, d, *J* = 7.8 Hz, 7.18, t, *J* = 7.2 Hz, 7.10, t, *J* = 7.2 Hz, 7.59, t, *J* = 7.8 Hz, 8.05, s (NH); δ_C_ 39.9 (t), 24.8 (t), 113.3 (s), 121.9 (d), 124.7 (s), 111.1 (d), 121.9 (d), 119.3 (d), 118.6 (d), and 127.6 (s)] were also observed. The 3-ethylindole group on the tertiary nitrogen in **5** was revealed by detailed analysis of 2D NMR spectra (Figure 8). The HMBC correlations of H-1′/C-11 (δ_C_ 170.2), C-8 (δ_C_ 137.2) as well as correlations of H-2′/C-3′, C-4′ and C-11′ indicated that the phenylethyl side chain at the nitrogen in compound **3** was replaced by the 3-ethylindole group in compound **5**. Assignment of the ^1^H and ^13^C-NMR spectroscopic data of **5** were accomplished by application of ^1^H–^1^H COSY, HMQC, and HMBC correlations. The relative configuration of compound **5** was assigned the same as those of compounds **3** and **4**.

Cespitaenin A (**6**) was isolated as a colorless, amorphous solid. The molecular formula, C_21_H_30_O_3_, was established by the HRESIMS at *m*/*z* 353.2096 [M + Na]^+^ (calcd. 353.2093). The IR bands at 1720 and 1706 cm^−1^ were attributed to an ester and a carbonyl group, which were confirmed by the presence of the acetate (δ_C_ 170.1) and ketocarbonyl (δ_C_ 202.1). The ^13^C-NMR (Table 2) and DEPT spectra of compound **6** revealed 21 carbons including three methyl carbons (δ_C_ 19.5, 24.8, and 32.8), six aliphatic methylene carbons (δ_C_ 30.6, 31.3, 41.1, 50.7, 23.8 and 22.8), a methine carbon (δ_C_ 43.1), an oxygenated methine carbon (δ_C_ 72.3), an aliphatic quaternary carbon (δ_C_ 35.4), two olefinic methine carbons (δ_C_ 129.0 and 135.4), an olefinic methylene carbon (δ_C_ 113.5), three olefinic quaternary carbons (δ_C_ 146.4, 133.3, and 148.0), and two additional carbonyl signals. The ^1^H–^1^H COSY spectrum showed the connectivities of H-7/H-6/H-5 and H-3/H-2/H-1/H-14/H-13/H-12. Resonances at δ_C_ 133.3 (C-8) and 129.0 (C-7) were correlated in the HMBC spectrum with proton signals at δ_H_ 5.15 (d, *J* = 8.4 Hz, H-7), and with the vinylic methyl protons at δ_H_ 1.76 (Me-19), and suggested that compound **6** contains an *E*-trisubstituted double bond bearing a methyl group [14]. In addition, a trisubstituted double bond [δ_C_ 148.0 (s), 135.4 (d), δ_H_ 6.30, t, *J* = 8.4 Hz] and a 1,1-disubstituted olefin (δ_C_ 144.7) with an exomethylene group (δ_C_ 115.5; δ_H_ 4.87, 4.95, each s) were also implied by interpretation of the HMBC data of compound **6**. Moreover, HMBC correlations of δ_H_ 5.38 (dt, *J* = 8.4, 2.4 Hz, H-6) with δ_C_ 170.1 indicated that C-6 (δ_C_ 72.3) is attached to an acetoxy group (δ_C_ 21.3). HMBC correlations of H-12/C-11, C-10, C-15, H-9/C-10, C-11, Me-16/C-11, C-15, C-1 and Me-17/C-11, C-15, C-1, H-18/C-3, C-5 established the final structure of **6**. The relative configuration of **6** was determined by NOESY analysis and comparison of the coupling constants of **6** with the data reported [14,15,16,17]. Assuming that H-1 is at the β position, the correlations between H-1/Me-16/Me-17 indicated the β-disposition of Me-16 and Me-17. The spin pattern and coupling constants of H-6, and NOESY correlations of H-6/Me-19/H-9α and H-7/H-9β agreed with a β-orientation of the acetoxy group at C-6.

**Figure 9:**
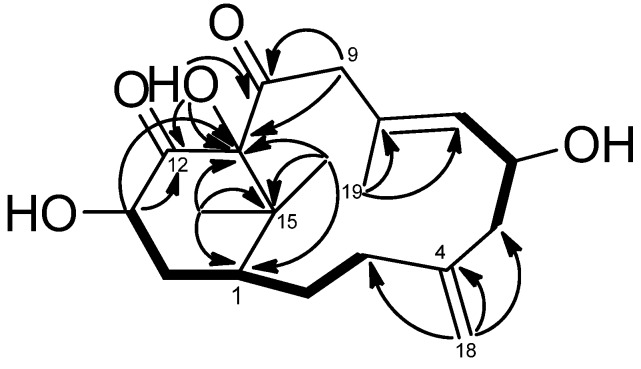
COSY (bold bond) and HMBC (arrow) correlations of **7**.

Cespitaenin B (**7**), ${\left[\alpha \right]}_{\text{D}}^{25}$ −109 (CH_2_Cl_2_), was isolated as a colorless, amorphous solid. Its molecular formula was determined to be C_19_H_28_O_5_ (Δ= 6) from HRESIMS at *m*/*z* 359.1837 [M + Na]^+^. Its IR bands showed the presence of a hydroxy (3397 cm^−1^) and conjugated carbonyl (1697 cm^−1^) groups. The ^1^H and ^13^C-NMR spectroscopic (Table 1 and Table 2) and DEPT data indicated the presence of two ketocarbonyls (δ_C_ 214.5 and 208.1), a trisubstituted olefin [δ_C_ 133.4 (s), 132.7 (d); δ_H_ 5.56, d, *J* = 9.3 Hz], and an exocyclic double bond [δ_C_ 144.8 (s), 115.5 (t); δ_H_ 4.92, 4.96, each s). In the aliphatic region, a quaternary carbon (δ_C_ 46.8), two oxygenated methine carbons (δ_C_ 70.2 and 74.8), an oxygenated tertiary carbon (δ_C_ 92.2), five methylene carbons (δ_C_ 32.9, 39.2, 46.8, 49.4, and 24.3), and three methyl groups (δ_C_ 25.8, 26.5, and 17.6; δ_H_ 0.77, 1.47, and 1.89, each s) were observed. HMQC correlations of δ_H_ 4.55 (dt, *J* = 9.6, 3.9 Hz, H-6) with δ_C_ 70.2 (d, C-6) and δ_H_ 4.39 (t, *J* = 3.3 Hz, H-13) with δ_C_ 74.8 (d, C-13) suggested that C-6 and C-13 are hydroxylated. The ^1^H–^1^H COSY spectrum indicated the connectivities of H-7/H-6/H-5 and H-3/H-2/H-1/H-14/H-13 to be similar with those of compound **6** (Figure 9). The two ketocarbonyls assigned at C-10 and C-12, and the hydroxyl group assigned at C-11 were deduced from the interpretation of HMBC correlations of H-9/C-10, C-11; H-13/C-12, C-11; Me-16, Me-17/C-1, C-11, C-15; OH-11 (δ_H_ 3.13, br s)/C-11, C-10, C-12. The remaining HMBC correlations of Me-16/C-15, C-1, Me-17/C-15, C-1 also indicated that compound **7** has the same 6/12 bicyclic system as compound **6**. The NOESY spectrum showed correlations of H-1/Me-16, Me-17, OH-11/Me-16 indicating that the hydroxy on C-11 is β*-*oriented, while H-6 is α*-*oriented due to the correlations of H-6/Me-19/H-9α (δ_H_ 3.89) and H-7/Me-17/H-9β (δ_H_ 2.84). The lack of correlations of H-13/H-1, Me-16, Me-17 was consistent with an α-orientation of H-13.

The molecular formula of cespitaenin C (**8**) was determined to be C_22_H_32_O_4_, as derived from a *quasi*-molecular ion at *m*/*z* 361.2378 ([M + Na]^+^, calcd. 361.2379), and seven indices of hydrogen deficiency. The IR spectrum displayed absorption bands suggestive of hydroxyl (3385 cm^−1^) and ester carbonyl (1738 cm^−1^) moieties. The ^1^H and ^13^C-NMR spectra (Table 1 and Table 2) exhibited an exomethylene double bond [δ_C_ 145.9 (s), 114.5 (t); δ_H_ 4.83, 4.84, each s], a trisubstituted double bond [δ_C_ 131.4 (s), 135.6 (d); δ_H_ 5.51, d, *J* = 7.8 Hz, H-7), a tetrasubstituted double bond (δ_C_ 166.6, C-11; 129.5, C-12), and an ester carbonyl (δ_C_ 170.5), accounting for four degrees of unsaturation. These findings implied that **8** is a tricyclic compound. The ^1^H–^1^H COSY correlations of H-7/H-6/H-5, H-3/H-2/H-1/H-14/H-13, and H-1′/H-2′, along with the HMBC correlations of H_2_-9/C-10, C-11, H-13/C-12, C-11, C-20; Me-16/C-11, C-12; Me-17/C-11, C-12 clearly indicated that compound **8** contains a common verticillene skeleton. HMBC correlations of H-1′/C-10 suggested the ethoxy group at C-10 and thus a carbonyl at C-20 (δ_C_ 170.5). The relative configuration of compound **8** was deduced from the NOESY analysis and comparison with chemical shifts and coupling constants of cespihypotin V [18]. The NOESY correlations of H-1′/Me-16, H-1/Me-17/Me-17 and H-6/Me-19 indicated that Me-16, Me-17, H-1, and the OEt were β-oriented, while H-6 is α-oriented.

**Figure 10:**
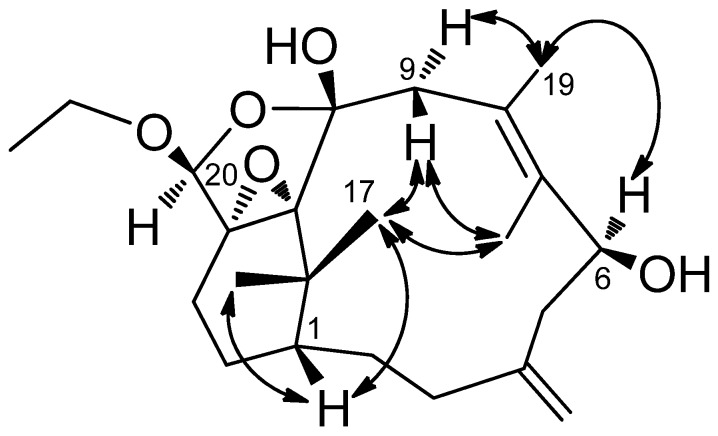
Selected NOESY correlation of **9**.

The HRESIMS data of cespitaenin D (**9**) established the molecular formula of C_22_H_34_O_5_ (*m*/*z* 401.2306, [M + Na]^+^), and indicated six indices of hydrogen deficiency. The IR spectrum displayed an absorption band indicative of hydroxy (3444 cm^−1^) group. The ^1^H and ^13^C-NMR spectroscopic data (Table 1 and Table 2) showed an exomethylene double bond (δ_C_ 145.8 (s), 115.6 (t); δ_H_ 4.92, s, 2H), a trisubstituted double bond [δ_C_ 133.2 (d), 132.8 (s); δ_H_ 5.45, d, *J* = 8.5 Hz, H-7), and a tetrasubstituted double bond, revealing two degrees of unsaturation. This implied that compound **9** possesses a tetracyclic ring system. The similar ^1^H, ^13^C-NMR, COSY, and HMBC data suggested that **9** should have the same verticillene skeleton as **8**. However, HMBC correlations of H-1′/C-20; H-13/C-12, C-11, C-20; H-20/C-12, C-11; Me-16, Me-17/C-11 indicated an ethoxy group at C-20 (δ_C_ 103.5) and an epoxy ring at C-11 (δ_C_ 72.8) and C-12 (δ_C_ 78.0). The epoxy ring at C-11 and C-12 was tentatively assigned the α-configuration due to the steric hindrance of the two β-faced methyl groups (Me-16 and Me-17). NOESY correlations (Figure 10) among H-1/Me-16, Me-17, H-6/Me-19/H-9α (δ_H_ 3.01) and H-7/H-9β (δ_H_ 2.53), and lack of NOESY correlation between H-20 and Me-17 indicated the β*-*orientation of the ethoxy group at C-20 and the α-disposition of H-6.

Cespitaenin E (**10**) was found to have the same molecular formula, C22H34O5, as **9**. It displayed as a sodium adduct ion at *m*/*z* 401.2305 ([M + Na]+) in the HRESIMS. There were very few differences between the 1H-NMR spectroscopic data (Table 1) of **9** and **10**. Comparison of their 13C-NMR spectra (Table 2) revealed that the differences occurred in the chemical shifts of C-13 (δC 26.0, **10**; 31.6, **9**) and C-20 (δC 107.3, **10**; 103.5, **9**). Furthermore, the COSY and HMBC correlations were closely comparable (Supporting Information). The NOESY correlations of H-20/Me-17 in **10** confirmed the β*-*orientation of H-20. The only difference between **9** and **10** is the configuration of the ethoxy group at C-20. The optical rotations of **10** [${\left[\alpha \right]}_{\text{D}}^{25}$ 0.1 (CH2Cl2)] and **9** [${\left[\alpha \right]}_{\text{D}}^{25}$ −20.6 (CH_2_Cl_2_)] supported the conclusion to be made that compound **10** is the 20-epimer of cespitaenin D.

**Scheme 1:**
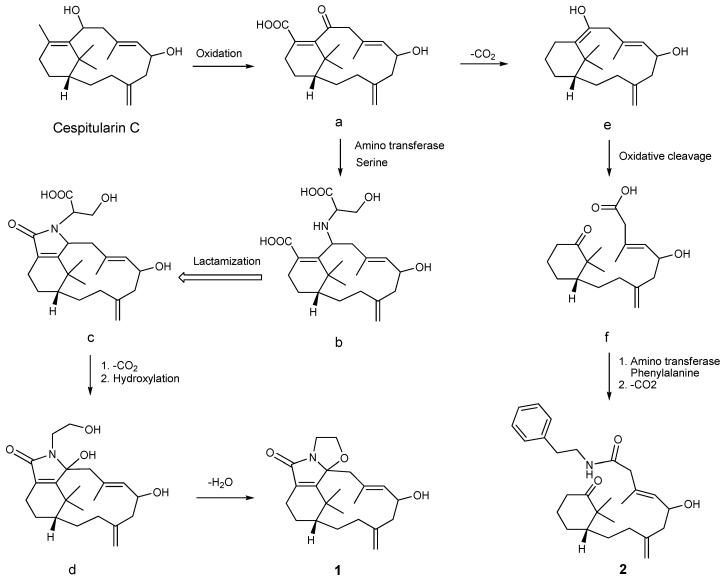
A postulated biosynthetic pathway for compounds **1** and **2**.

A postulated biosynthetic pathway for compounds **1** and **2** is illustrated in Scheme 1. Compound **1** is probably produced from cespitularin C [19] via intermediates **a**–**d**, involving steps of oxidation, serine transformation, lactamization, decarboxylation, hydroxylation, and dehydration. Compound **2** may be generated from the nor-verticillene **a** through intermediates **e** and **f**. These reactions deal with decarboxylation, cleavage of the double bond between C-10 and C-11 [19], and phenylalanine transformation leading to an amide formation.

Four human cancer cell lines were chosen to test the *in vitro* cytotoxicity of compounds **1**–**10** (Table 3). Compound **5** exhibited cytotoxicity against human breast adenocarcinoma (MCF-7), medulloblastoma (Daoy), and cervical epitheloid carcinoma (Hela) cancer cells with IC_50_ of 17.5, 22.3, and 24.7 μM, respectively. Compound **6** showed significant cytotoxicity against human breast adenocarcinoma (MCF-7) cancer cells with the IC_50_ at 21.2 μM.

**Table 3:** Cytotoxicity of compounds **1**–**10** against human cancer cells (IC_50_, μM) *^a^.*

Compound	Hela	Daoy	WiDr	MCF-7
**3**	30.9	34.8	49.5	30.6
**5**	24.7	22.3	34.1	17.5
**6**	28.5	31.5	36.4	21.2
mitomycin C	0.32	0.32	0.32	0.32

*^a^* Hela: human cervical epitheloid carcinoma; Daoy: human medulloblastoma; WiDr: Human colon adenocarcinoma; MCF-7: human breast adenocarcinoma; *^b^* Compounds **1**, **2**, **4**, **7**–**10** were inactive (>40 μM) in this assay system.

## 3. Experimental Section

### 3.1. General Experimental Procedures

Optical rotations were obtained on a JASCO DIP-1000 polarimeter. IR spectra were recorded using a Horiba FT-720 spectrophotometer. The ^1^H and ^13^C-NMR spectra as well as 2D NMR spectra (^1^H–^1^H COSY, HSQC, HMBC, and NOESY) were recorded in CDCl_3_ (or CD_3_OD) using Bruker DRX NMR spectrometers operating at 300 or 500 MHz for ^1^H and 75 or 125 MHz for ^13^C using the CDCl_3_ solvent peak as internal standard (δ_H_ 7.26 for ^1^H and δ_C_ 77.0 for ^13^C). Low-resolution ESIMS and HRESIMS were run on a JEOL JMS-HX 110 mass spectrometer. Silica gel 60 (Merck, Darmstadt, Germany) was used for column chromatography (CC). Precoated silica gel plate (Kieselgel 60 F-254, 1 mm, Merck, Darmstadt, Germany) was used for preparative TLC. Sephadex LH-20 (Amersham Pharmacia Biotech AB, Uppsala, Sweden) was used for separation. LiChrospher Si 60 (5 μm, 250-10, Merck, Darmstadt, Germany) and LiChrospher 100 RP-18e (5 μm, 250-10, Merck, Darmstadt, Germany) were used for NP-HPLC and RP-HPLC (Hitachi, Tokyo, Japan), respectively.

### 3.2. Animal Material

*Cespitularia taeniata* was collected in Green Island, Taiwan, in March 2004. This soft coral was identified by one of the authors (Y.-C.S.). A voucher specimen (GSC-1) has been deposited in the School of Pharmacy, National Taiwan University, Taipei, Taiwan.

### 3.3. Extraction and Isolation

The whole animals of *C. taeniata* (dried, 1.1 kg) were extracted with EtOAc and CH_2_Cl_2_ (1:1, each 1 L × 3) at room temperature and concentrated under reduced pressure to yield a crude extract. The crude extract was partitioned between H_2_O and EtOAc to yield an EtOAc-soluble fraction (100 g), which was chromatographed on a Si gel column (1 kg) and initially eluted with *n*-hexane (100%, 1 L), *n*-hexane/EtOAc (15:1 to 0:1, each 1 L), and finally MeOH (100%, 1 L) to give 12 fractions. Fractions six (3.1 g) and eight (1.7 g) were further separated on a Sephadex LH-20 column using CH_2_Cl_2_-MeOH (4:1) to furnish nine and five fractions (6-1~6-9, 8-1~8-5), respectively. Separation of fraction 6-5 (390 mg) was performed by a Si gel column (1.2 g) using a solvent mixture of *n*-hexane-CH_2_Cl_2_-MeOH (100:100:1~5:5:1) to yield six fractions (6-2-1~6-2-6). Fraction 6-5-3 (34 mg) was further purified with a NP-HPLC column (*n*-hexane-CH_2_Cl_2_-MeOH, 15:15:1) to give cespitaenin A (**6**, 2 mg). Fraction 6-5-4 (121 mg) and fraction 6-5-5 (68 mg) were separated with a NP-HPLC column (CH_2_Cl_2_-MeOH, 80:1) and then a RP-HPLC column was used (MeOH-H_2_O-CH_3_CN, 70:25:5) to yield cespitaenin C (**8**, 6 mg), cespitaenin D (**9**, 6 mg), cespilamide C (**3**, 5 mg) and cespitaenin E (**10**, 2.5 mg). Fraction 6-6 (310 mg) was purified with a NP-HPLC column (CH_2_Cl_2_-MeOH, 80:1) and with preparative TLC (*n*-hexane-BuOH, 12:1) to give cespilamide D (**4**, 9 mg). Fraction 6-8 (16 mg) was further purified with a RP-HPLC column (MeOH-H_2_O-CH_3_CN, 70:25:5) to yield cespilamide E (**5**, 5 mg). Fraction 8-4 (779 mg) and 8-5 (68 mg) were further separated with a NP-HPLC column (*n*-hexane-CH_2_Cl_2_-MeOH, 20:20:1) and with a RP-HPLC column (MeOH-H_2_O-CH_3_CN, 65:30:5) to yield cespilamide A (**1**, 1.5 mg), cespitaenin B (**7**, 3 mg) and cespilamide B (**2**, 3 mg).

### 3.4. Spectral Data

Cespilamide A (**1**): colorless, amorphous solid; ${\left[\alpha \right]}_{\text{D}}^{25}$ −118 (*c* 0.2, CH_2_Cl_2_); IR (neat) ν_max_ 3421, 2936, 1695 cm^−1^; ^1^H-NMR (CDCl_3_, 300 MHz) and ^13^C-NMR (CDCl_3_, 75 MHz) data, see Table 1 and Table 2, respectively; HRESIMS *m*/*z* 358.2380 ([M + Na]^+^, calcd for C_22_H_31_O_3_NNa^+^, 358.2382).

Cespilamide B (**2**): colorless, amorphous solid; ${\left[\alpha \right]}_{\text{D}}^{25}$ −8.2 (*c* 0.2, CH_2_Cl_2_); IR (neat) ν_max_ 3371, 2929, 2360, 1701, 1647 cm^−1^; ^1^H-NMR (CDCl_3_, 500 MHz) and ^13^C-NMR (CDCl_3_, 125 MHz) data, see Table 1 and Table 2, respectively; HRESIMS *m*/*z* 448.2825 ([M + Na]^+^, calcd for C_27_H_39_O_3_NNa^+^, 448.2827).

Cespilamide C (**3**): colorless, amorphous solid; ${\left[\alpha \right]}_{\text{D}}^{25}$ 15.5 (*c* 0.2, CH_2_Cl_2_); IR (neat) ν_max_ 2926, 1676 cm^−1^; ^1^H-NMR (CDCl_3_, 300 MHz) and ^13^C-NMR (CDCl_3_, 75 MHz) data, see Table 1 and Table 2, respectively; HRESIMS *m*/*z* 356.1992 ([M + Na]^+^, calcd for C_23_H_27_ONNa^+^, 356.1990).

Cespilamide D (**4**): colorless, amorphous solid; ${\left[\alpha \right]}_{\text{D}}^{25}$ 18.2 (*c* 0.2, CH_2_Cl_2_); IR (neat) ν_max_ 3312, 2927, 1649 cm^−1^; ^1^H-NMR (CDCl_3_, 300 MHz) and ^13^C-NMR (CDCl_3_, 75 MHz) data, see Table 1 and Table 2, respectively; HRESIMS *m*/*z* 372.1937 ([M + Na]^+^, calcd for C_21_H_30_O_3_Na^+^, 372.1939).

Cespilamide E (**5**): colorless, amorphous solid; ${\left[\alpha \right]}_{\text{D}}^{25}$ 23.6 (*c* 0.2, CH_2_Cl_2_); IR (neat) ν_max_ 2929, 1659, 1340 cm^−1^; ^1^H-NMR (CDCl_3_, 300 MHz) and ^13^C-NMR (CDCl_3_, 75 MHz) data, see Table 1 and Table 2, respectively; HRESIMS *m*/*z* 395.2099 ([M + Na]^+^, calcd for C_25_H_28_ON_2_Na^+^, 395.2099).

Cespitaenin A (**6**): colorless, amorphous solid; ${\left[\alpha \right]}_{\text{D}}^{25}$ 9.7 (*c* 0.2, CH_2_Cl_2_); IR (neat) ν_max_ 1720, 1706 cm^−1^; ^1^H-NMR (CDCl_3_, 300 MHz) and ^13^C-NMR (CDCl_3_, 75 MHz) data, see Table 1 and Table 2, respectively; HRESIMS *m*/*z* 353.2096 ([M + Na]^+^, calcd for C_21_H_30_O_3_Na^+^, 353.2093).

Cespitaenin B (**7**): colorless, amorphous solid; ${\left[\alpha \right]}_{\text{D}}^{25}$ −109 (*c* 0.2, CH_2_Cl_2_); IR (neat) ν_max_ 3397, 2359, 2339, 1697, 1276 cm^−1^; ^1^H-NMR (CDCl_3_, 300 MHz) and ^13^C-NMR (CDCl_3_, 75 MHz) data, see Table 1 and Table 2, respectively; HRESIMS *m*/*z* 359.1837 ([M + Na]^+^, calcd for C_19_H_28_O_5_ Na^+^, 359.1834).

Cespitaenin C (**8**): colorless, amorphous solid; ${\left[\alpha \right]}_{\text{D}}^{25}$ −35.5 (*c* 0.2, CH_2_Cl_2_); IR (neat) ν_max_ 3385, 2924, 1738 cm^−1^; ^1^H-NMR (CDCl_3_, 300 MHz) and ^13^C-NMR (CDCl_3_, 75 MHz) data, see Table 1 and Table 2, respectively; HRESIMS *m*/*z* 361.2378 ([M + Na]^+^, calcd for C_22_H_32_O_4_Na^+^, 361.2379).

Cespitaenin D (**9**): colorless, amorphous solid; ${\left[\alpha \right]}_{\text{D}}^{25}$ 0.1 (*c* 0.2, CH_2_Cl_2_); IR (neat) ν_max_ 3444, 2986, 2950, 1731 cm^−1^; ^1^H-NMR (CDCl_3_, 500 MHz) and ^13^C-NMR (CDCl_3_, 125 MHz) data, see Table 1 and Table 2, respectively; HRESIMS *m*/*z* 401.2306 ([M + Na]^+^, calcd for C_22_H_34_O_5_Na^+^, 401.2304).

Cespitaenin E (**10**): colorless, amorphous solid; ${\left[\alpha \right]}_{\text{D}}^{25}$ −20.6 (*c* 0.2, CH_2_Cl_2_); IR (neat) ν_max_ 3390, 2930, 1757 cm^−1^; ^1^H-NMR (CDCl_3_, 300 MHz) and ^13^C-NMR (CDCl_3_, 75 MHz) data, see Table 1 and Table 2, respectively; HRESIMS *m*/*z* 401.2305 ([M + Na]^+^, calcd for C_22_H_34_O_5_Na^+^, 401.2304).

### 3.5. Preparation of (S)- and (R)-MPTA Esters (**1a** and **1b**) from **1**

*R*-(−)- or *S*-(+)-MPTA chloride (one drop) was added to a solution of **1** (3 mg in 2 mL pyridine) and the solution was allowed to stand at room temperature for 12 h. After purification using preparative LC, the resultant ester (3 mg, 90% yield) was analyzed by ^1^H NMR spectroscopic measurement, and ∆ = δ*_S_* − δ*_R_* was calculated. *Compound **1a**: *^1^H-NMR (CDCl_3_, 300 MHz) δ_H_ 5.578 (1H, dd, *J* = 8.9, 7.2 Hz, H-6), 5.542 (1H, overlap, H-7), 1.199, 1.466 (6H, s, H-16, 17), 4.788 (1H, s, H-18), 4.770 (1H, s, H-18), 1.597 (3H, s, H-19), 4.12 (1H, t, *J* = 6.6 Hz, H-1′), 3.92 (1H, t, *J* = 6.6 Hz, H-1′), 3.89 (1H, m, H-2″), 3.25 (1H, m, H-2″); *Compound **1b**:* ^1^H-NMR (CDCl_3_, 300 MHz) δ_H_ 5.525 (1H, dd, *J* = 8.9, 7.2 Hz, H-6), 5.402 (1H, d, *J* = 8.9 Hz, H-7), 1.189, 1.444 (6H, s, H-16, 17), 4.871 (1H, s, H-18), 4.835 (1H, s, H-18), 1.586 (3H, s, H-19), 4.12 (1H, t, *J* = 6.6 Hz, H-1′), 3.92 (1H, t, *J* = 6.6 Hz, H-1′), 3.87 (1H, m, H-2″), 3.25 (1H, m, H-2″).

### 3.6. Cytotoxicity Assay

Cytotoxicity was tested against the MCF-7 (breast carcinoma), Daoy (medulloblastoma), DLD-1 (colon adenocarcinoma), and Hela (cervical epitheloid adenocarcinoma) human tumor cell lines. The assay procedure using MTT [3-(4,5-dimethylthiazole-2-yl)-2,5-diphenyltetrazolium bromide was carried out as previously described.[20] The cells were cultured in RPMI-1640 medium. After seeding of the cells in a 96-well microplate for 4 h, 20 µL of sample was placed in each well and incubated at 37 °C for three days, and then 20 µL MTT was added and allowed to stand for 5 h. Then the medium was removed and DMSO (200 µL/well) was added and the mixture was shaken for 10 min. The formazan crystals were redissolved and their absorbance was measured on a microtiter plate reader (MR 7000, Dynatech, Scottsdale, USA) at a wavelength of 550 nm. The ED_50_ value was defined by a comparison with the untreated cells as the concentration of test sample resulting in 50% reduction of absorbance. Mitomycin C was used as the positive control.

## 4. Conclusions

This paper describes the first isolation of five novel nitrogen-containing diterpenoids and sesquiterpenoids, and five bicyclic verticillenes and nor-verticillenes from Taiwanese soft coral *Cespitularia taeniata*.

## Supplementary Files

Supplementary File 1
