# Supplementary material for: Nitrogen-Containing Diterpenoids, Sesquiterpenoids, and Nor-Diterpenoids from Cespitularia taeniata
Source: Mar Drugs. 2015 Sep 15;13(9):5796–814. doi: 10.3390/md13095796 (PMC4584355; doi:10.3390/md13095796)
Supplement: Supplementary File 1 [file marinedrugs-13-05796-s001.docx]

Supplementary Information


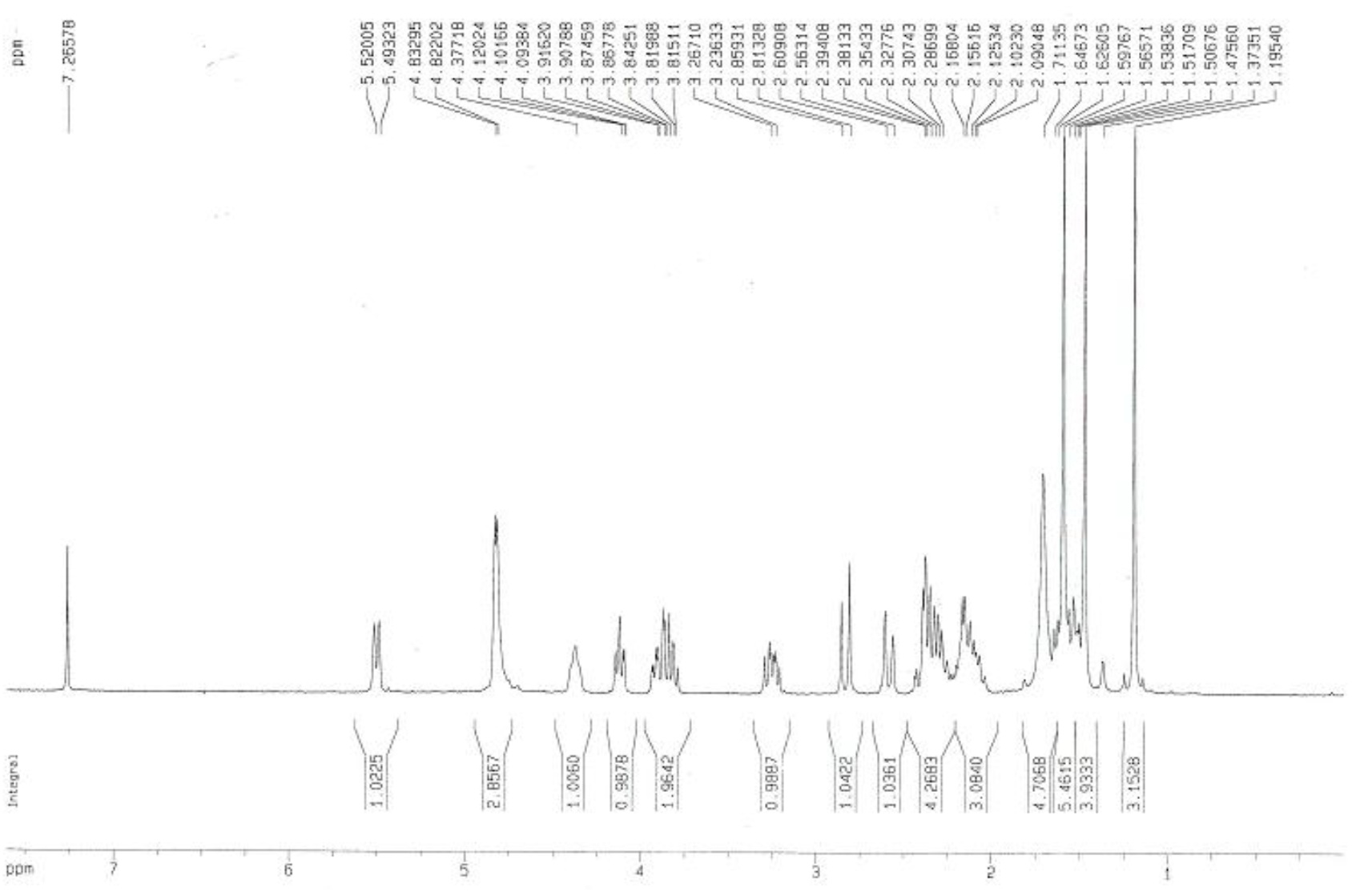


**Figure S1.** ^1^H NMR spectrum (300 MHz, CDCl_3_) of Cespilamide A (**1**).


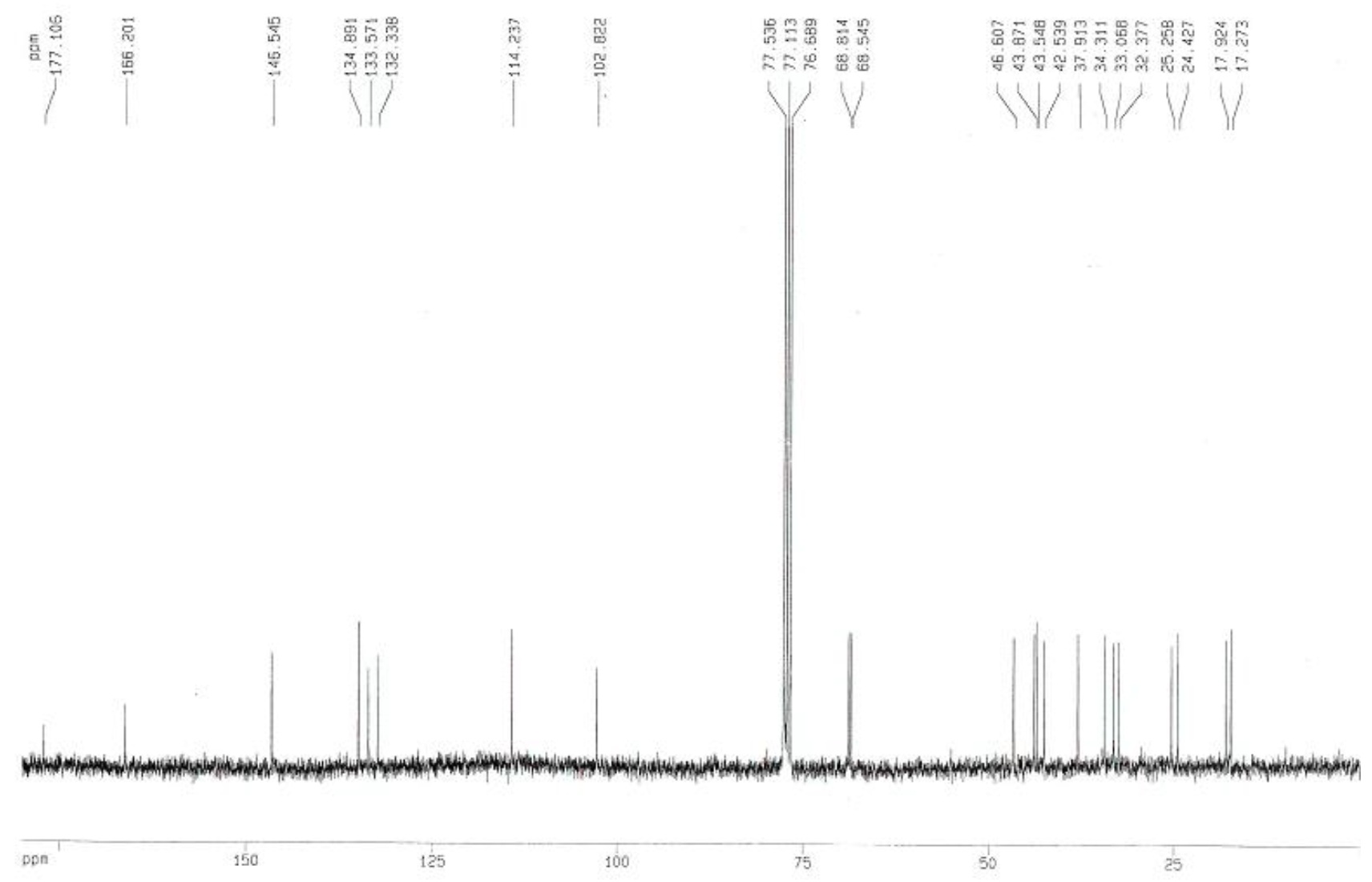


**Figure S2.** ^13^C NMR spectrum (75 MHz, CDCl_3_) of Cespilamide A (**1**).


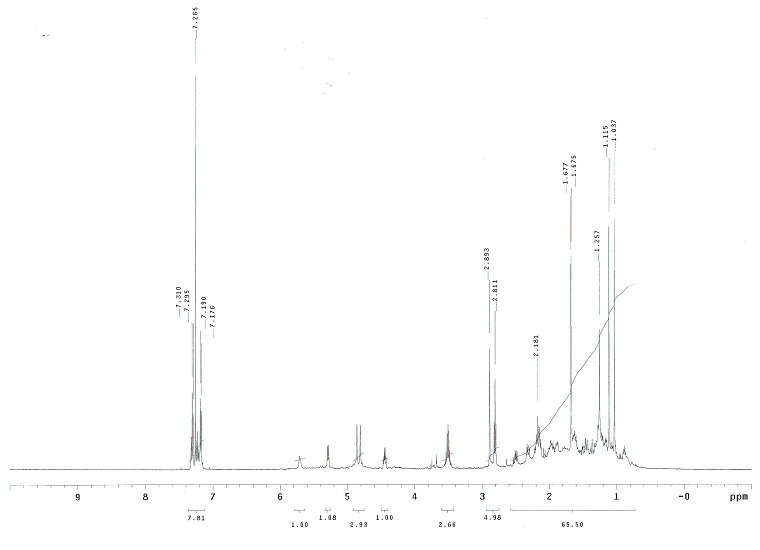


**Figure S3.** ^1^H NMR spectrum (500 MHz, CDCl_3_) of Cespilamide B (**2**).


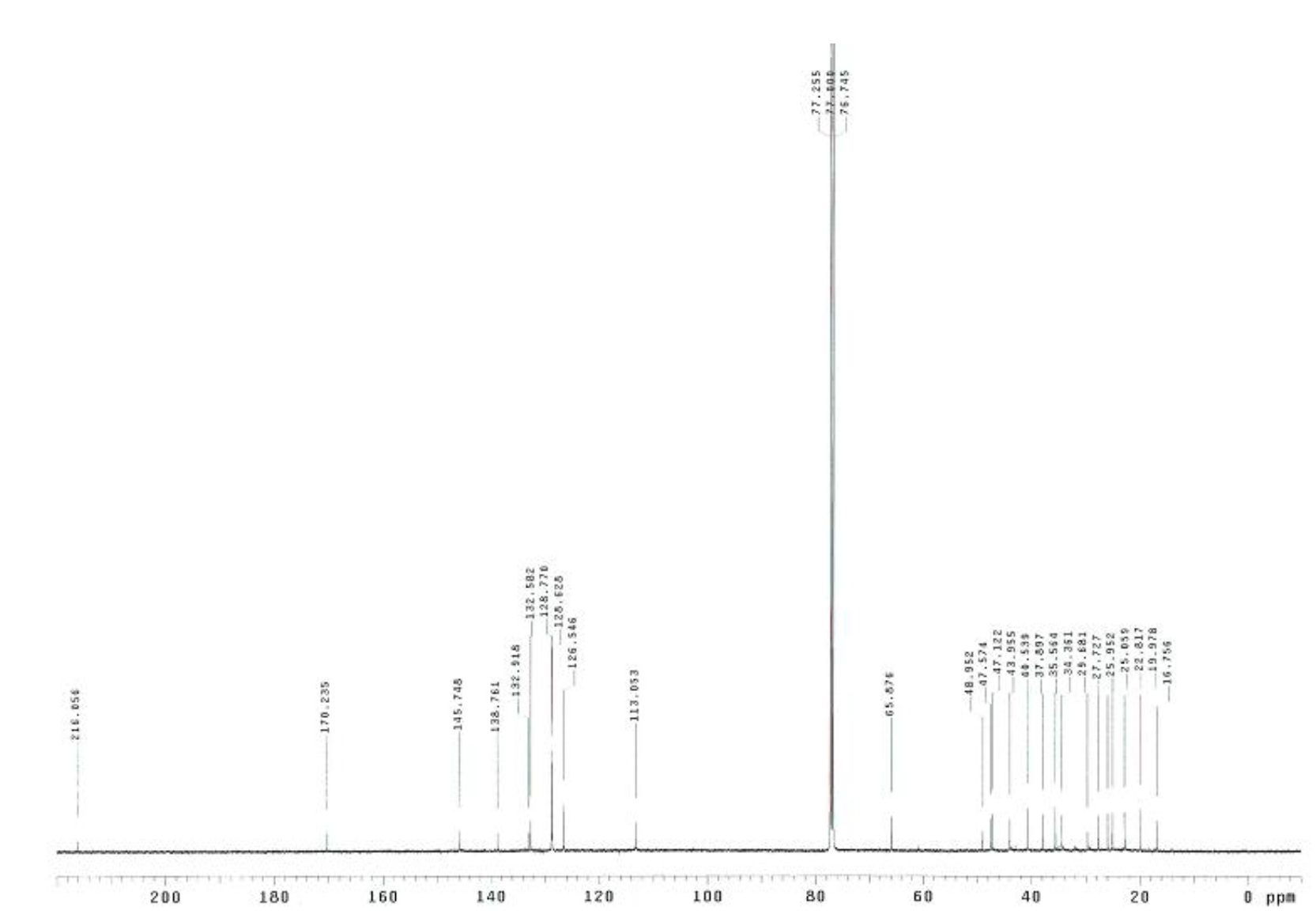


**Figure S4.** ^13^C NMR spectrum (125 MHz, CDCl_3_) of Cespilamide B (**2**).


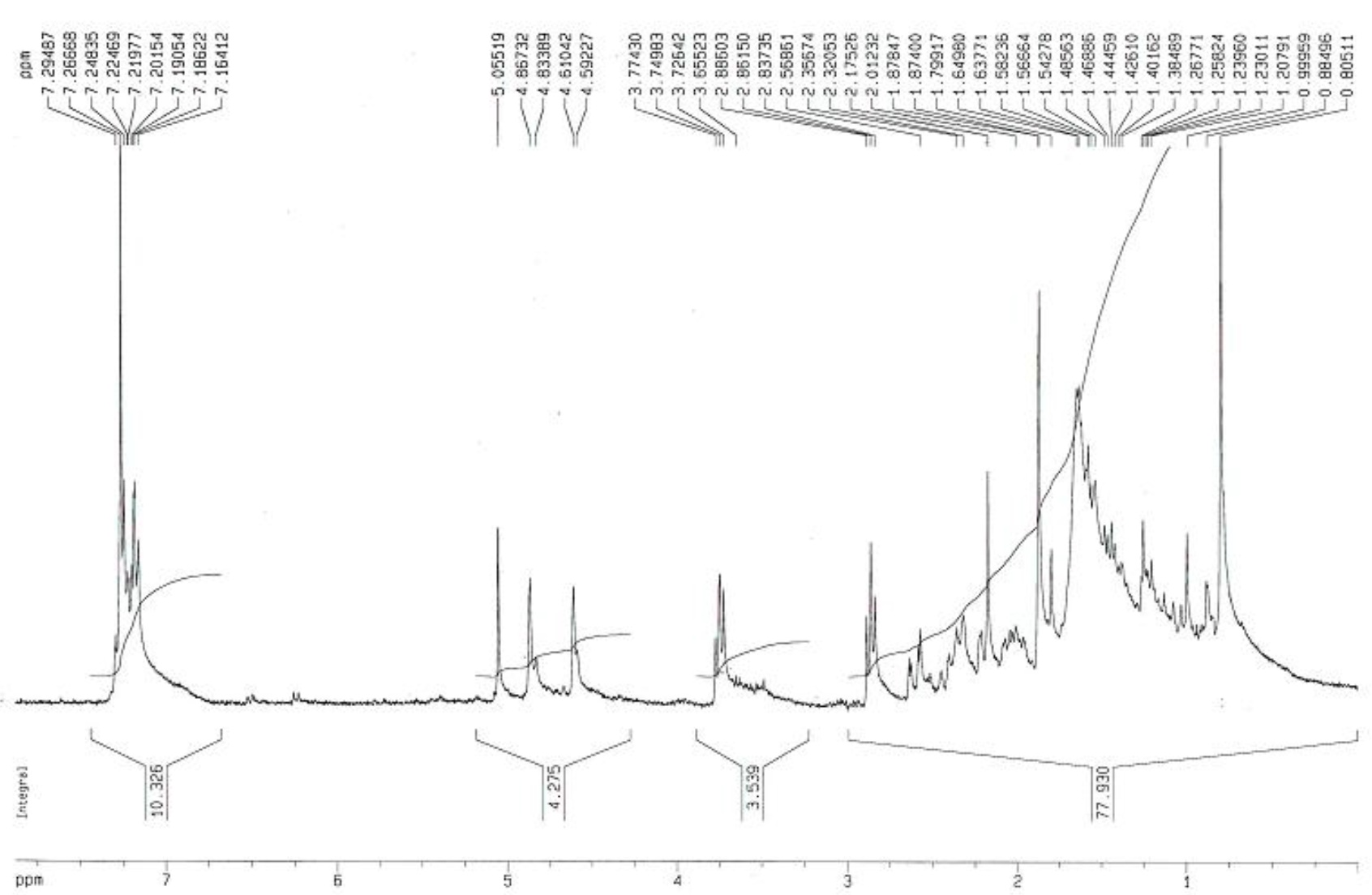


**Figure S5.** ^1^H NMR spectrum (300 MHz, CDCl_3_) of Cespilamide C (**3**).


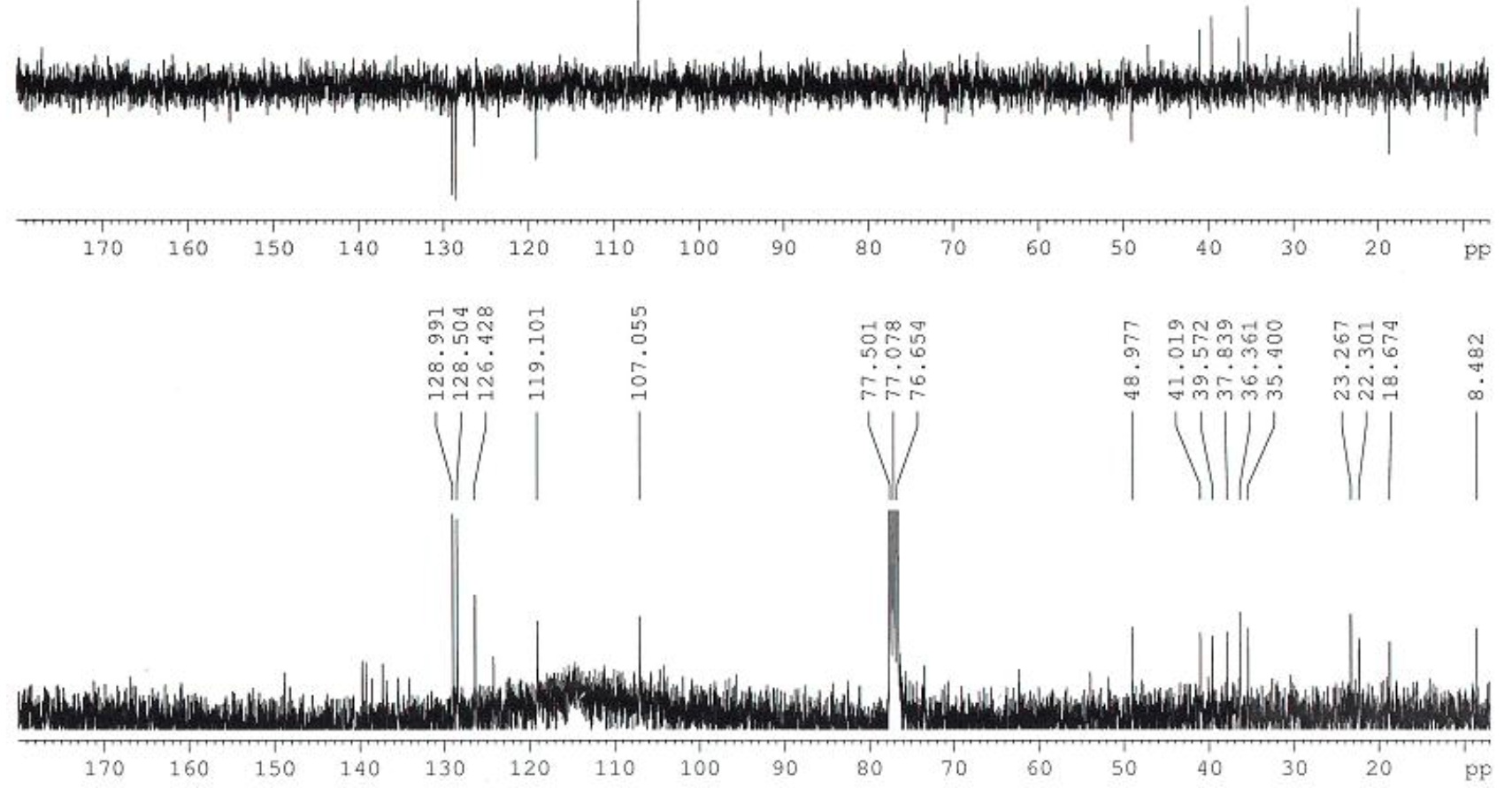


**Figure S6.** ^13^C NMR spectrum (75 MHz, CDCl_3_) of Cespilamide C (**3**).


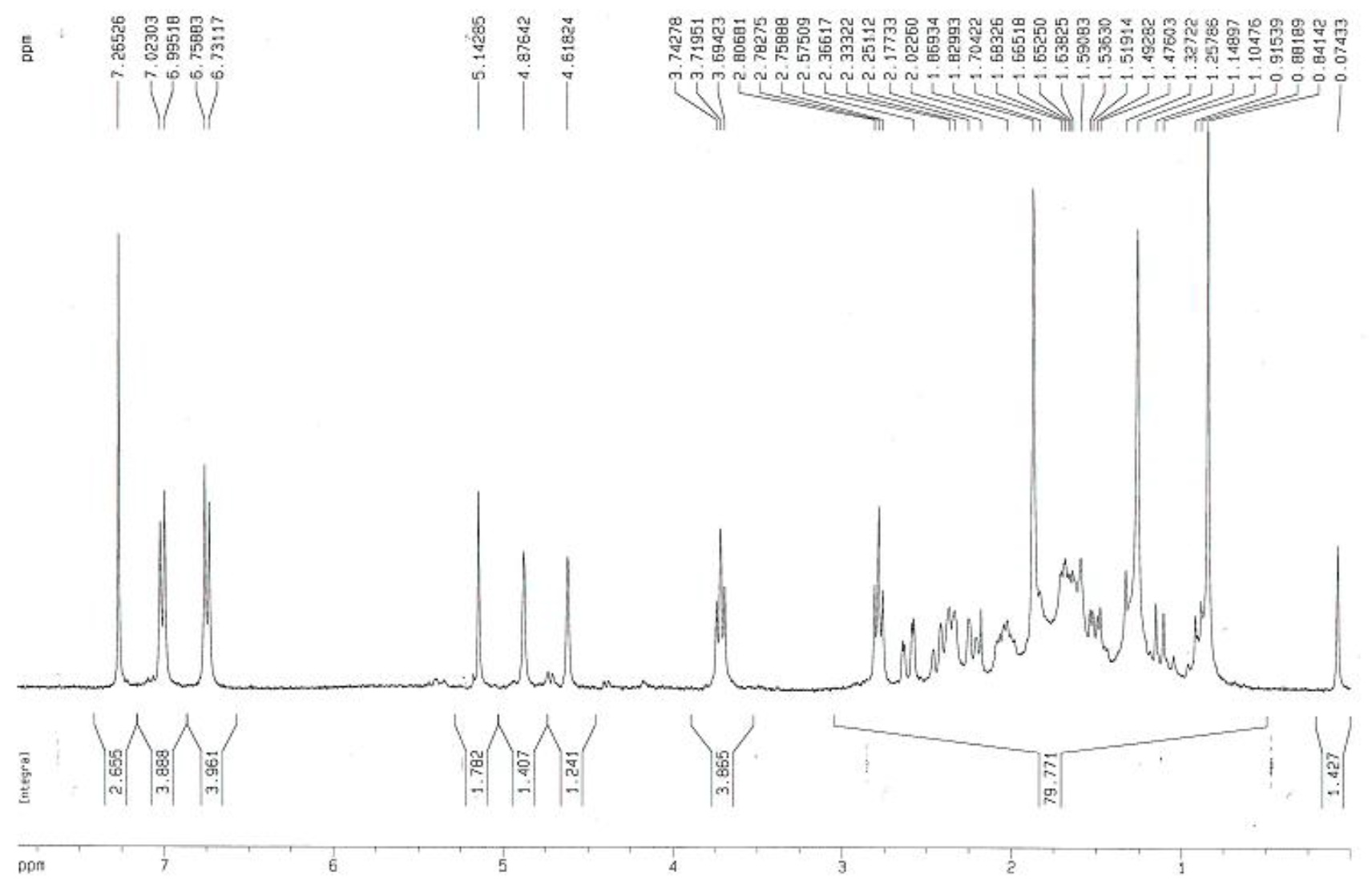


**Figure S7.** ^1^H NMR spectrum (300 MHz, CDCl_3_) of Cespilamide D (**4**).


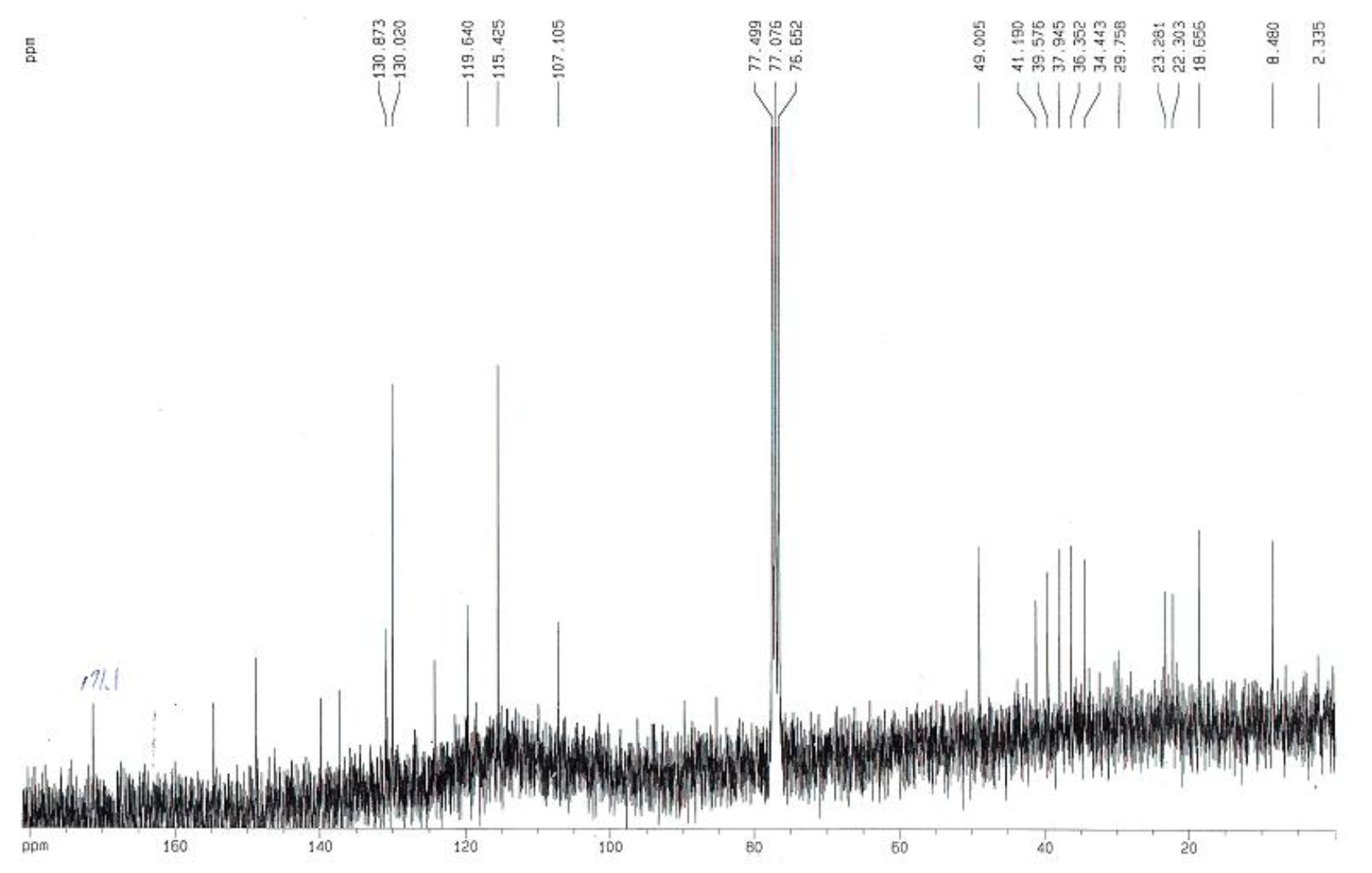


**Figure S8.** ^13^C NMR spectrum (75 MHz, CDCl_3_) of Cespilamide D (**4**).


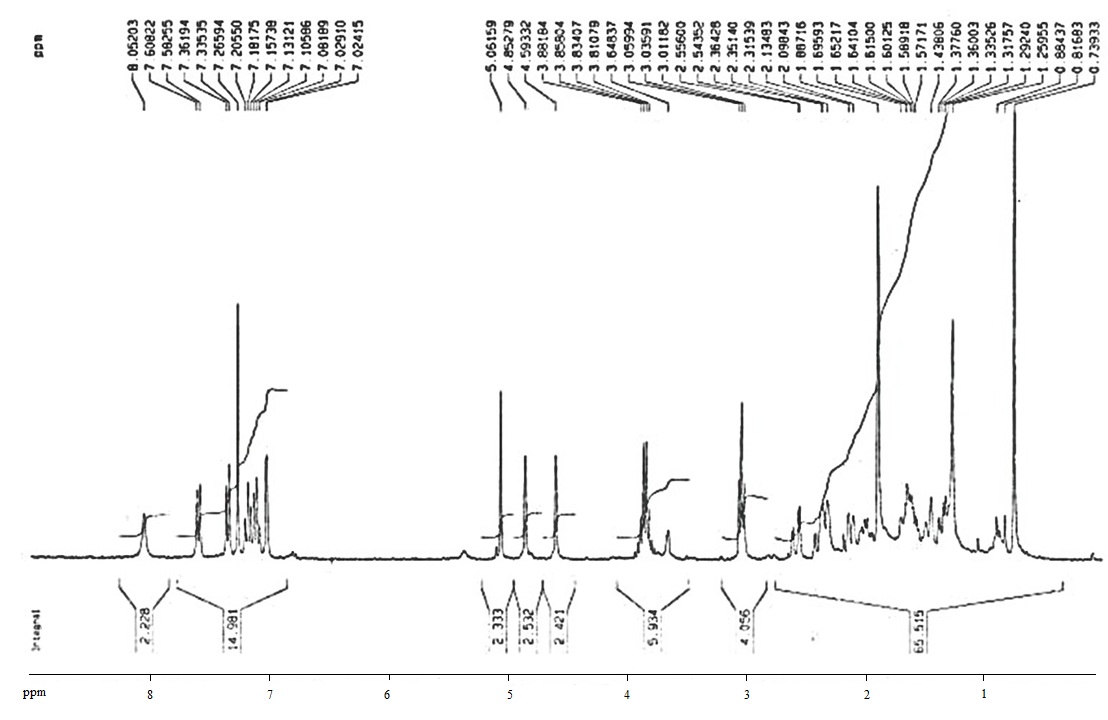


**Figure S9.** ^1^H NMR spectrum (300 MHz, CDCl_3_) of Cespilamide E (**5**).


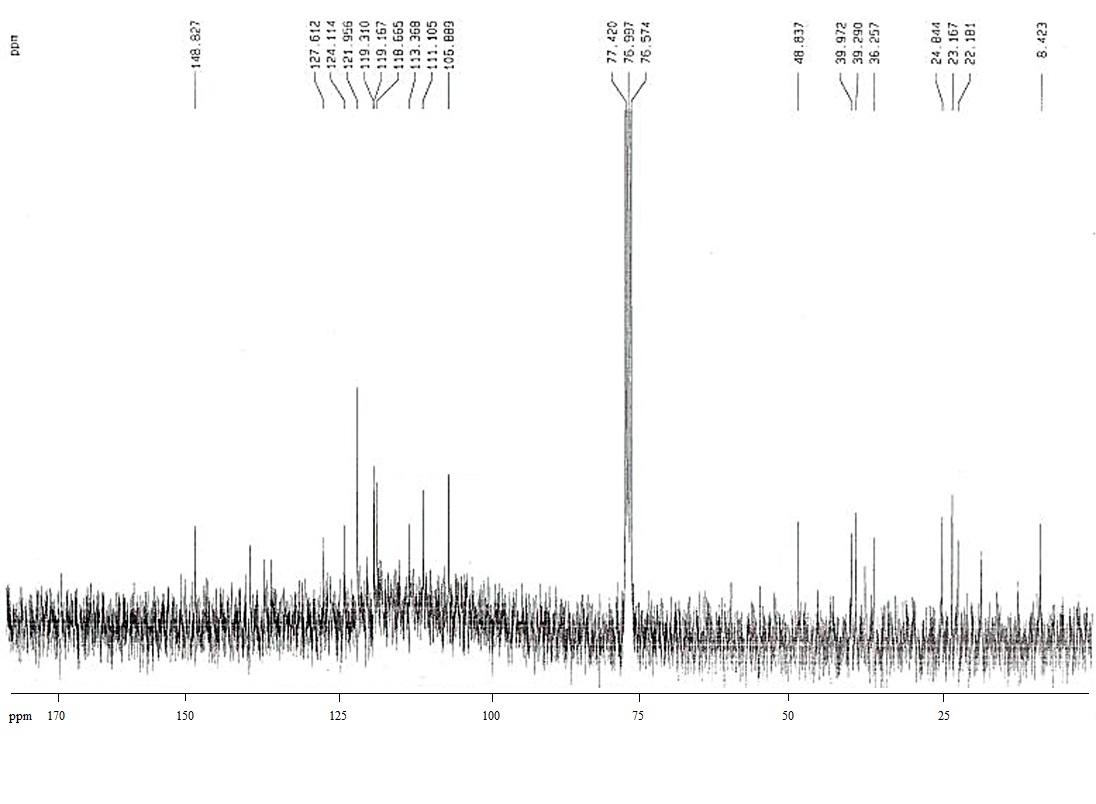


**Figure S10.** ^13^C NMR (75 MHz, CDCl_3_) of Cespilamide E (**5**).


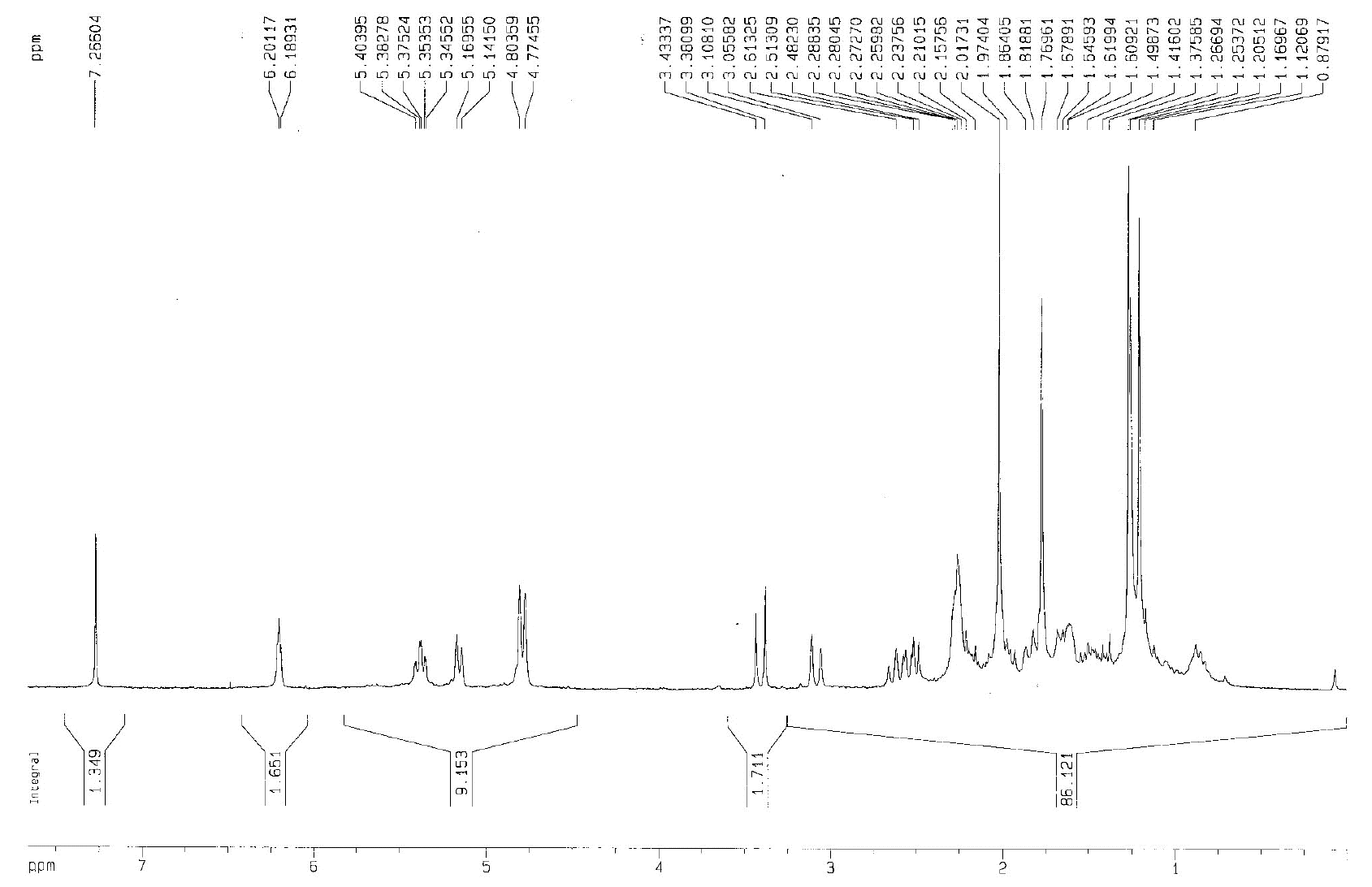


**Figure S11.** ^1^H NMR (300 MHz, CDCl_3_) of Cespitaenin A (**6**).


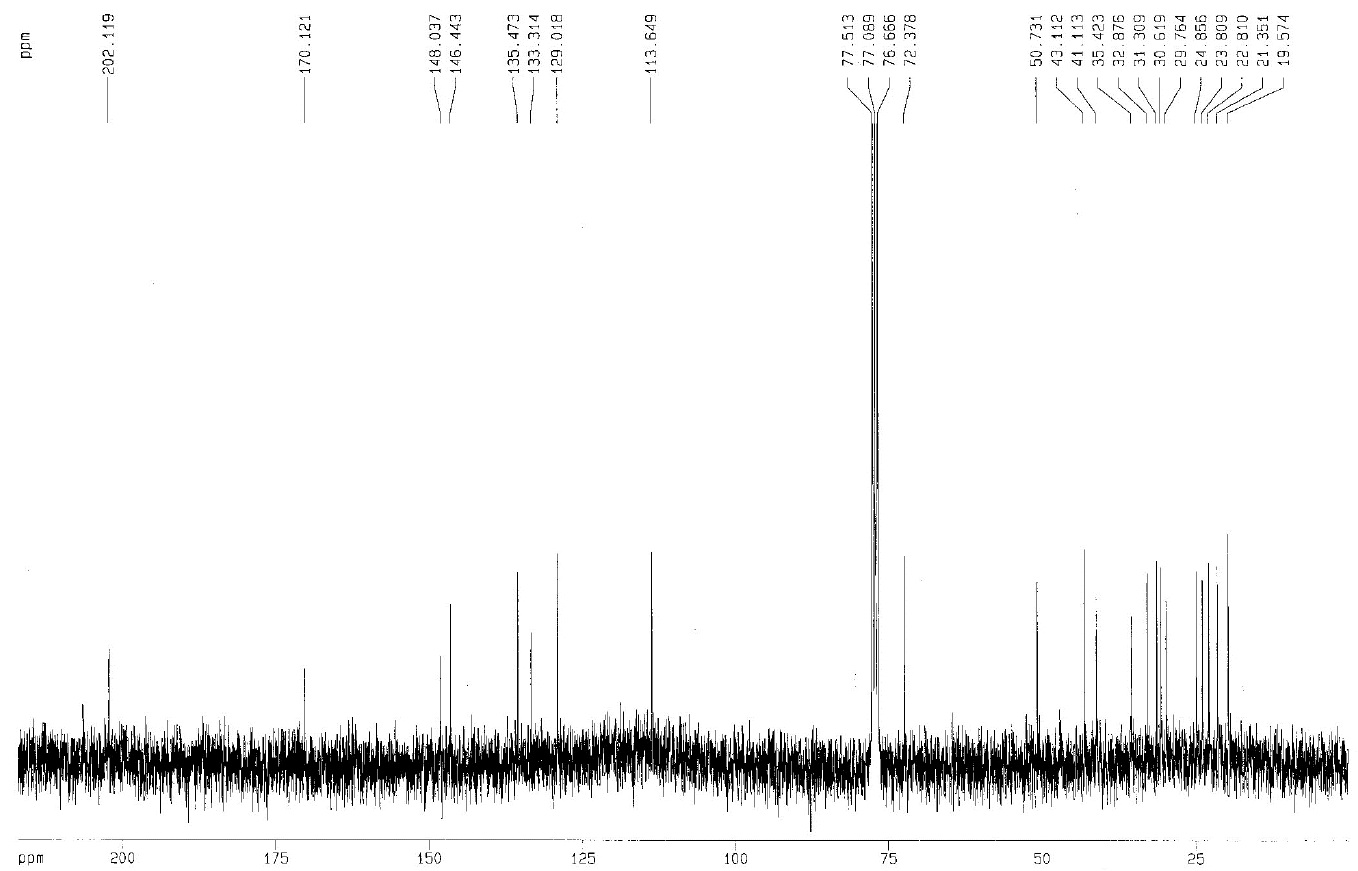


**Figure S12.** ^13^C NMR (75 MHz, CDCl_3_) of Cespitaenin A (**6**).


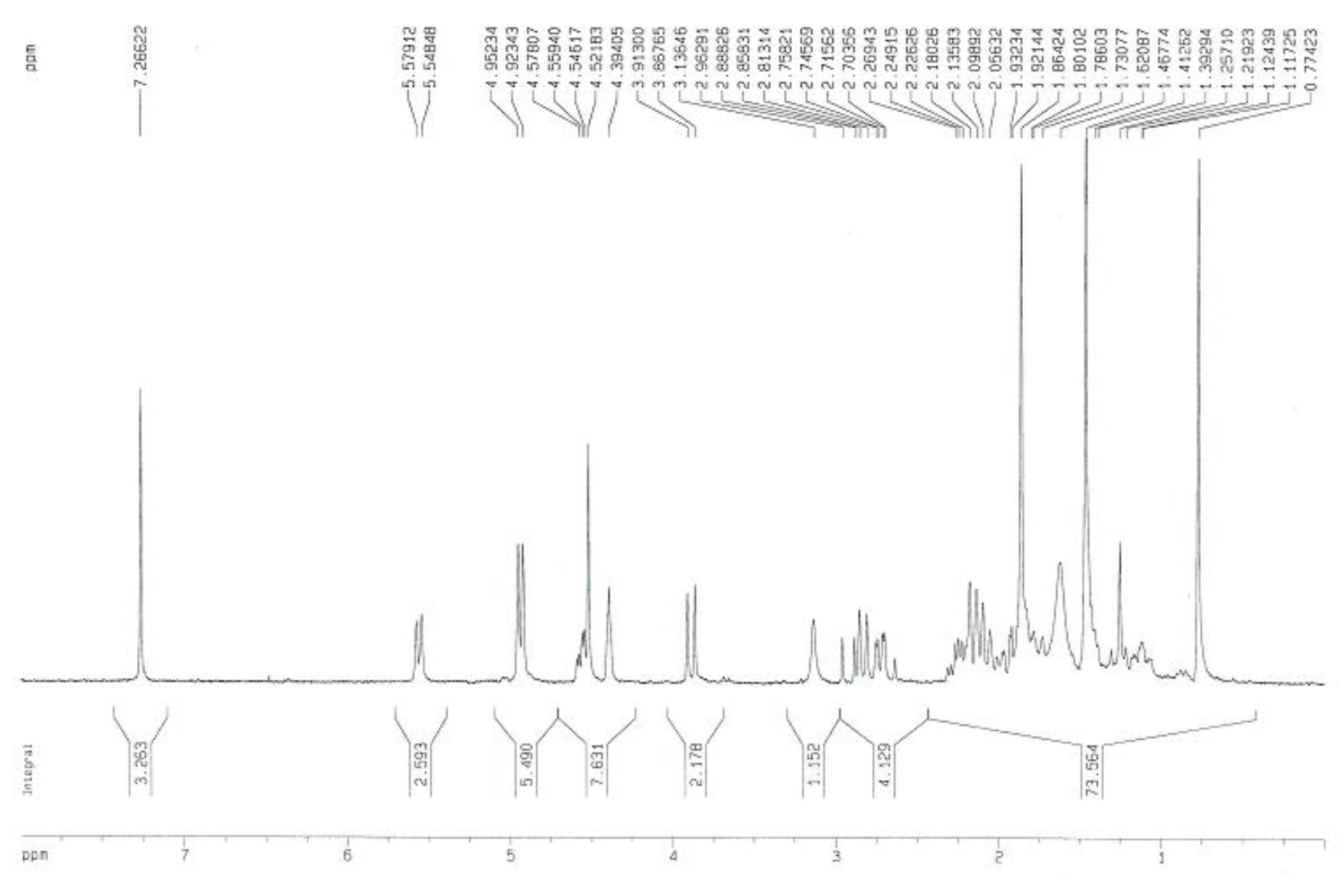


**Figure S13.** ^1^H NMR (300 MHz, CDCl_3_) of Cespitaenin B (**7**).


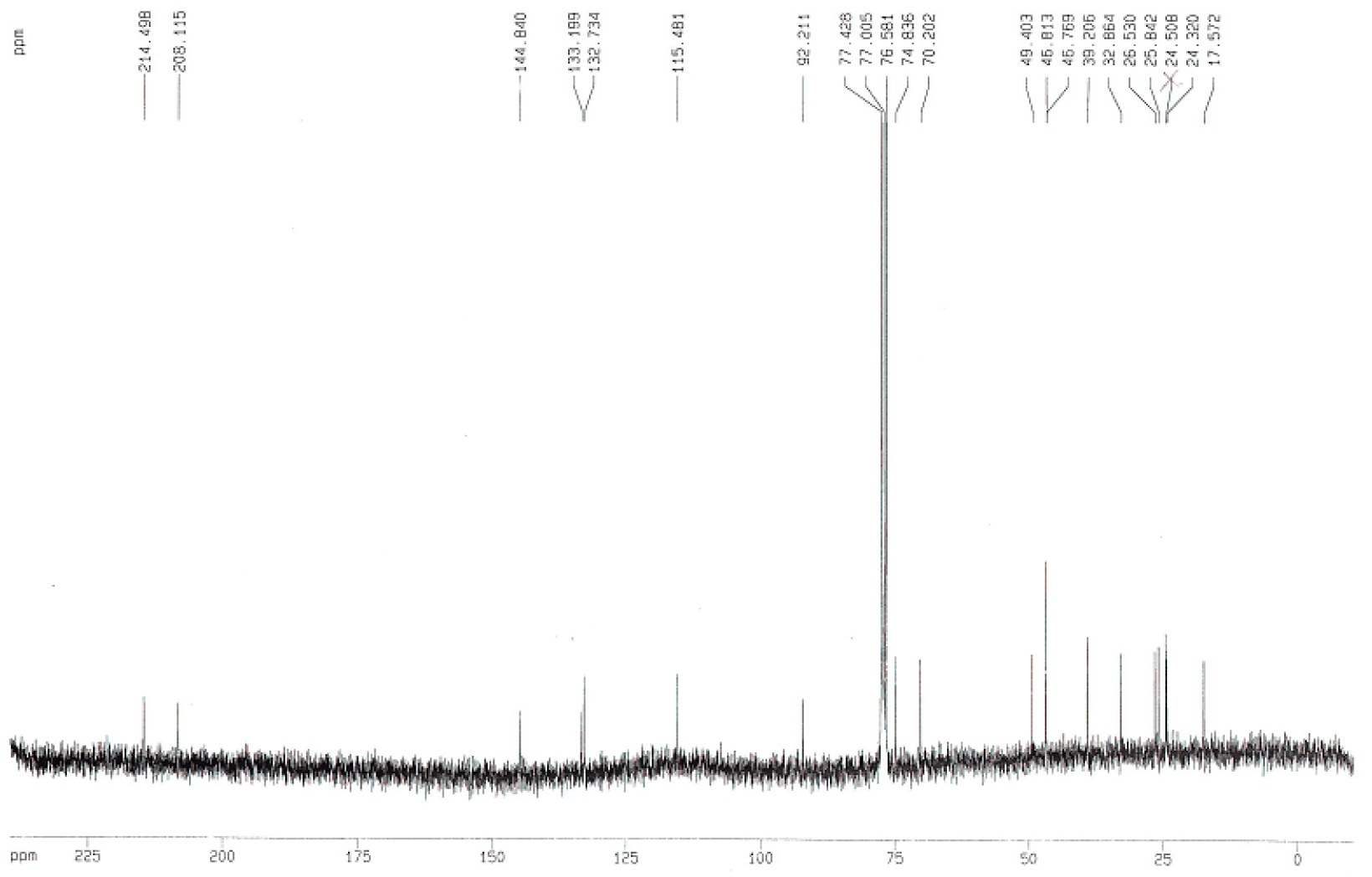


**Figure S14.** ^13^C NMR (75 MHz, CDCl_3_) of Cespitaenin B (**7**).


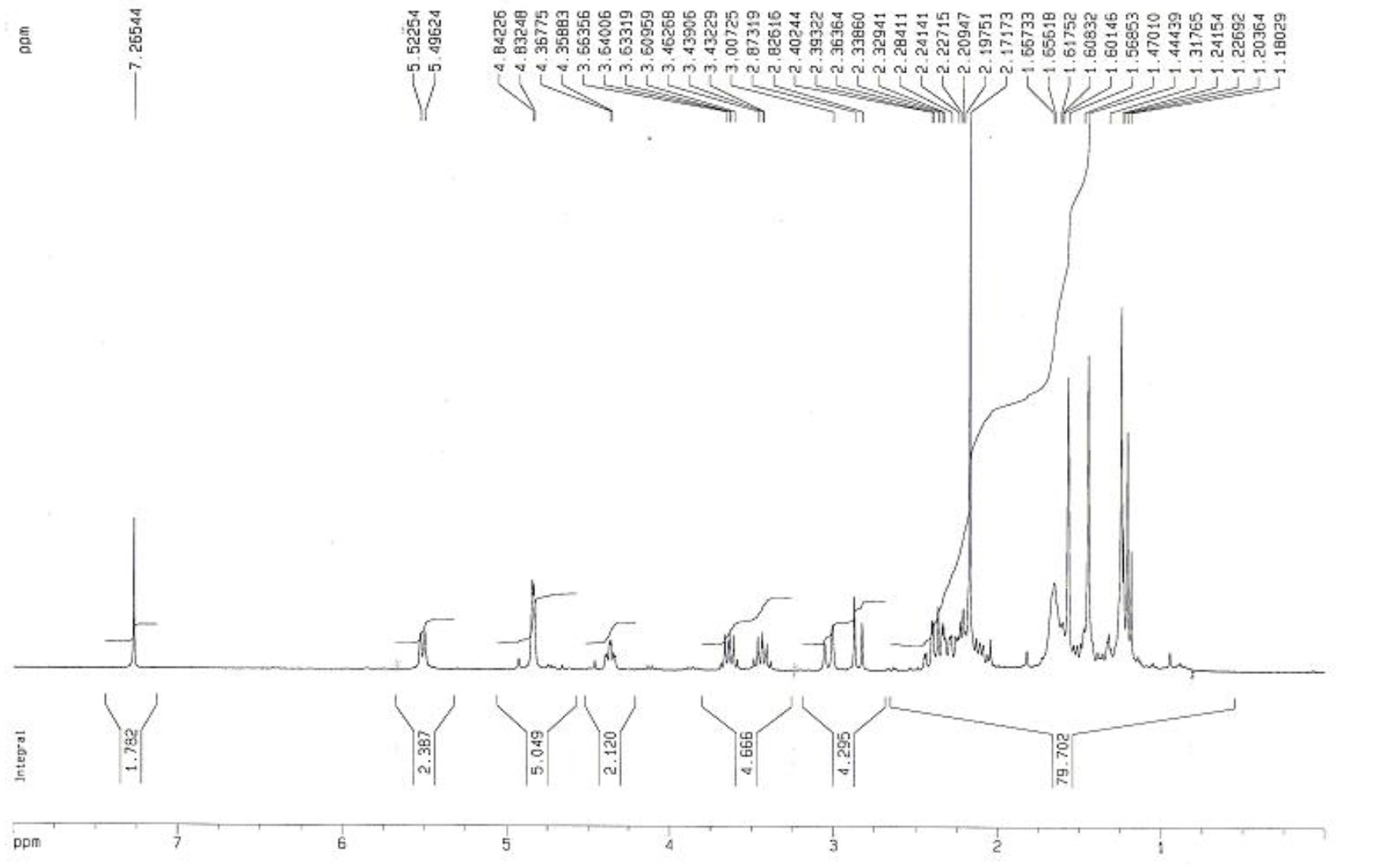


**Figure S15.** ^1^H NMR (300 MHz, CDCl_3_) of Cespitaenin C (**8**).


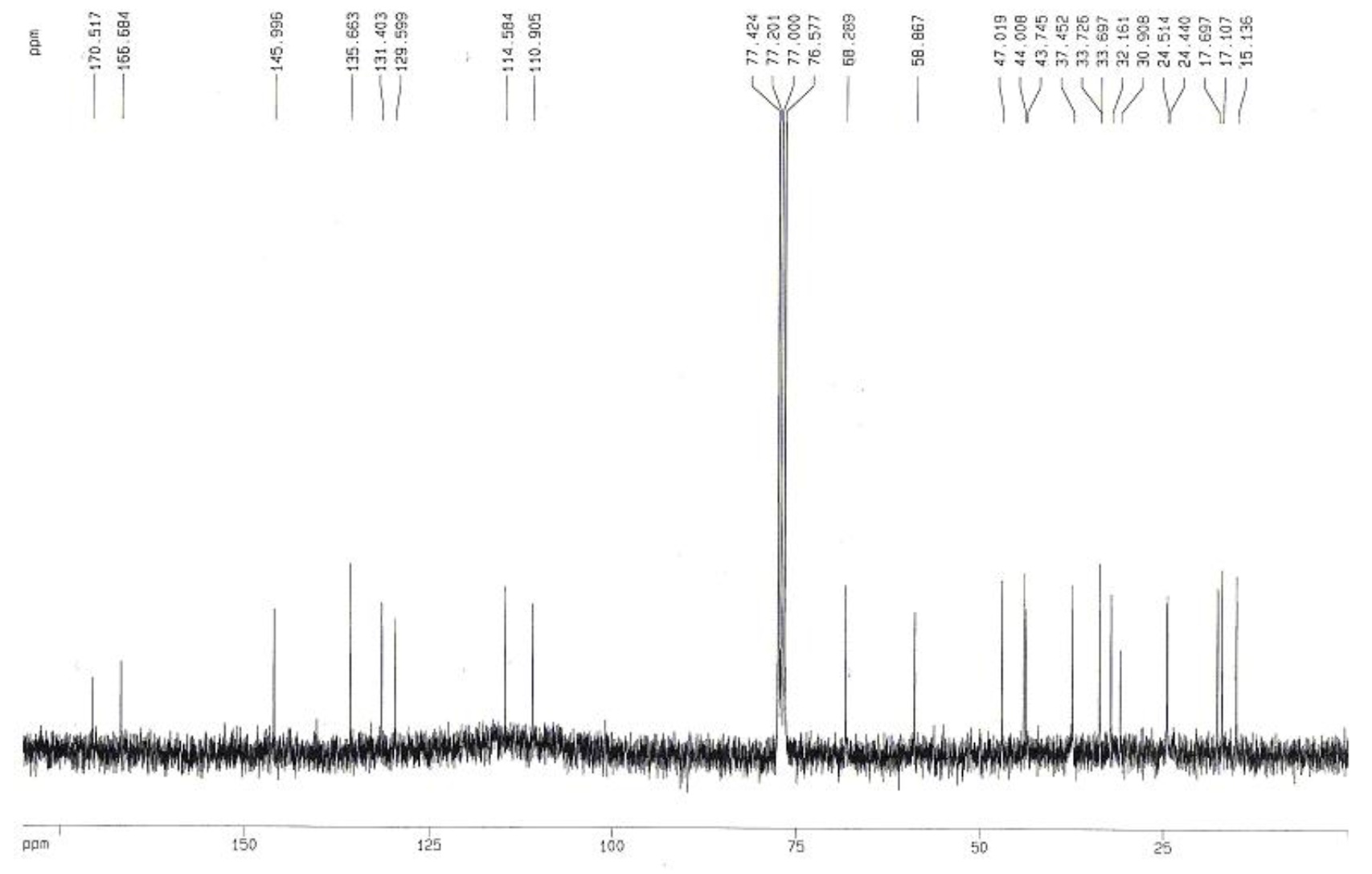


**Figure S16.** ^13^C NMR (75 MHz, CDCl_3_) of Cespitaenin C (**8**).


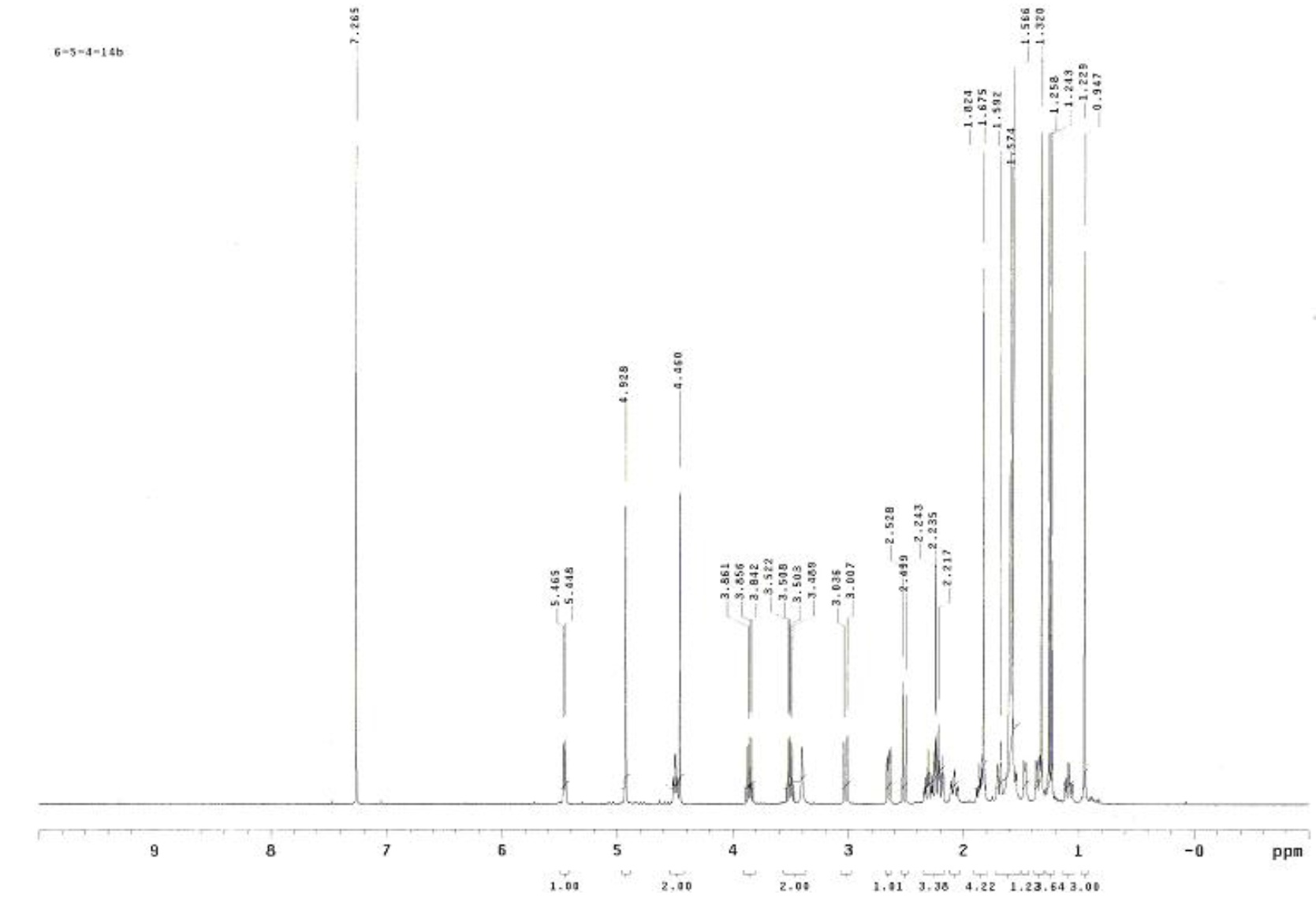


**Figure S17.** ^1^H NMR (500 MHz, CDCl_3_) of Cespitaenin D (**9**).


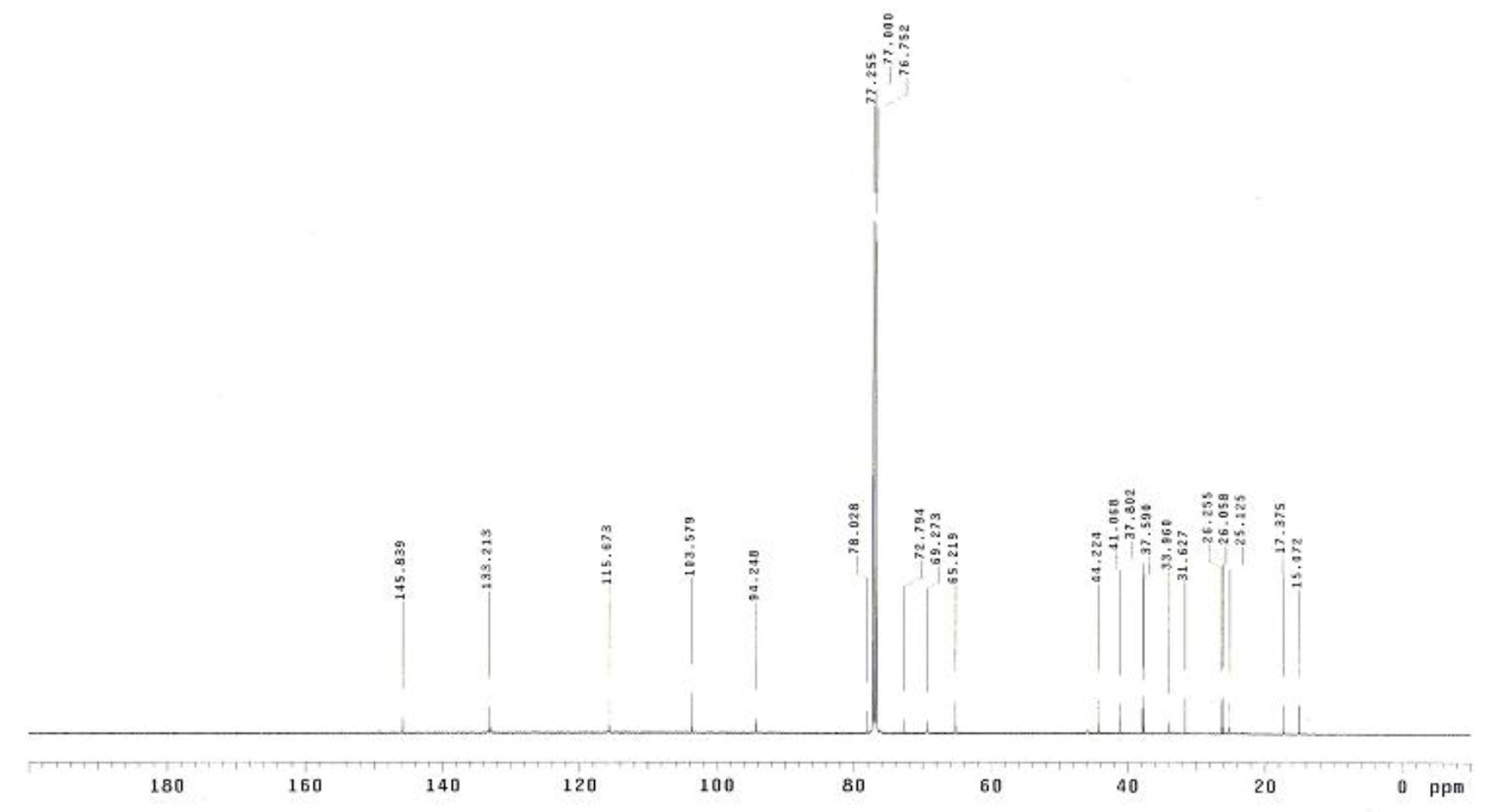


**Figure S18.** ^13^C NMR (125 MHz, CDCl_3_) of Cespitaenin D (**9**).


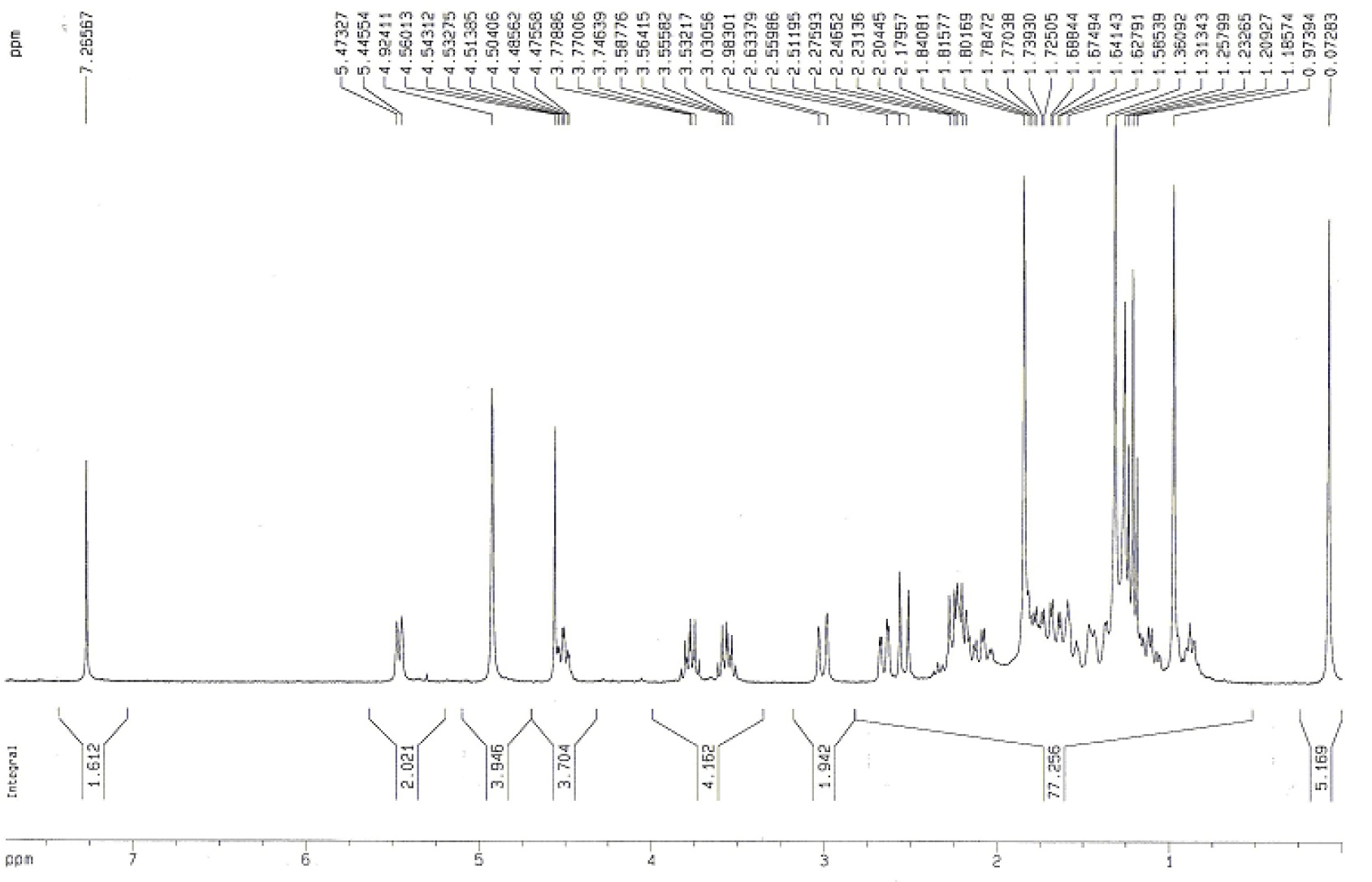


**Figure S19.** ^1^H NMR (300 MHz, CDCl_3_) of Cespitaenin E (**10**).


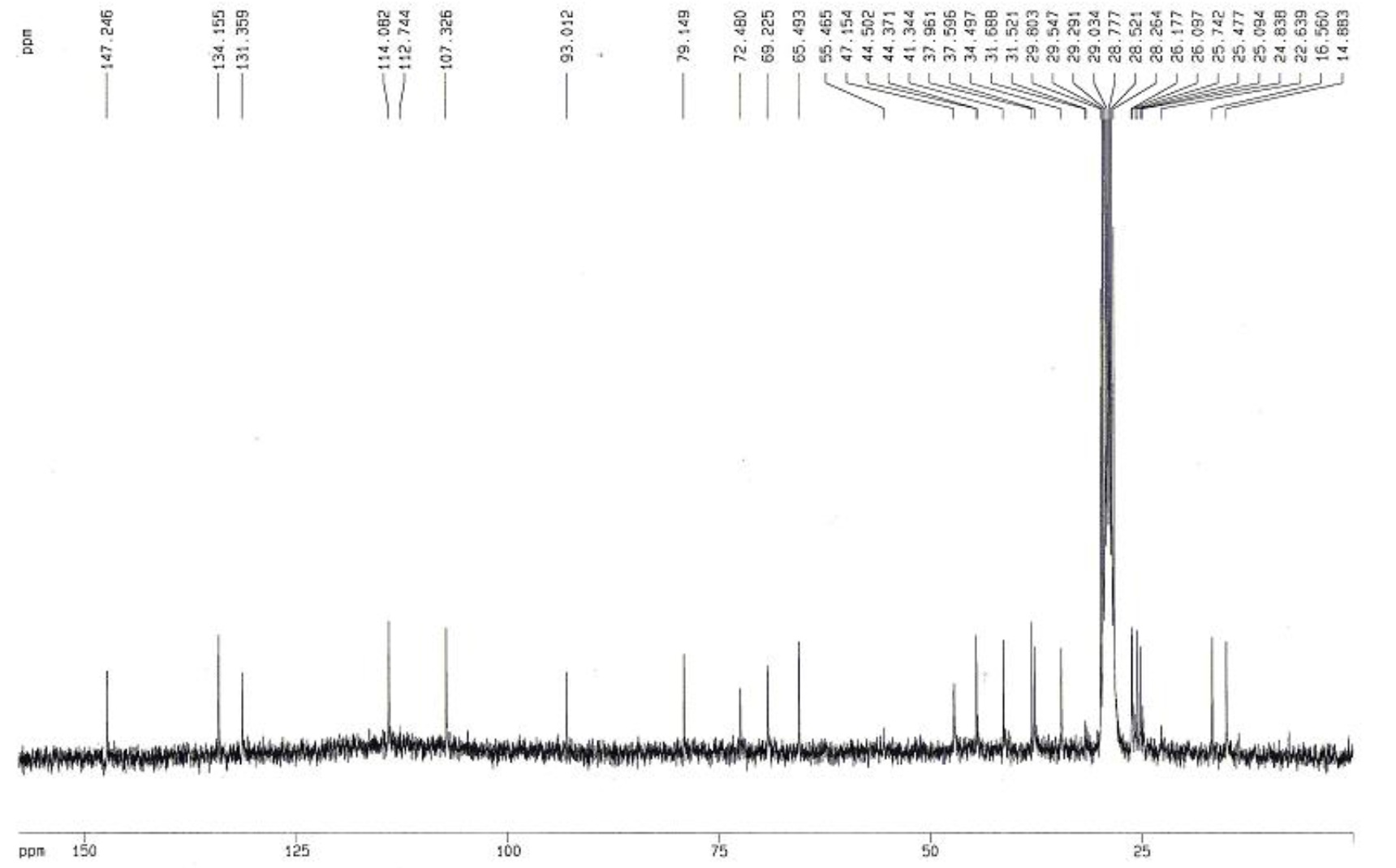


**Figure S20.** ^13^C NMR (75 MHz, CDCl_3_) of Cespitaenin E (**10**).

© 2015 by the authors; licensee MDPI, Basel, Switzerland. This article is an open access article distributed under the terms and conditions of the Creative Commons Attribution license (http://creativecommons.org/licenses/by/4.0/).
